# Integrated experimental and life cycle assessment of biodiesel production from *Datura stramonium* seed oil using a one-pot sulfonated corncob catalyst

**DOI:** 10.1038/s41598-026-52881-1

**Published:** 2026-06-10

**Authors:** Workisa Bacha Garuma, Tesfaye Kassaw Bedru, Gadissa Tokuma Gindaba, Abdi Nemera Emana, Edosa Toyi, Mani Jayakumar, Beteley Tekola Meshesha, Shegaw Ahmed Mohammed, Abayneh Getachew Demesa, Samuel Perez Vega, Guedri Okba

**Affiliations:** 1 Department of Chemical Engineering, Haramaya Institute of Technology, Haramaya University, P. O. Box 138, Haramaya, Dire Dawa, Ethiopia; 2 Department of Chemical Engineering, Kombolcha Institute of Technology, Wollo University, P.O. Box 208, Kombolcha, Ethiopia; 3 Department of Biotechnology, Faculty of Engineering, Karpagam Academy of Higher Education, Coimbatore, Tamilnadu 641 021 India; 4 Centre for Natural Products and Functional Foods, Karpagam Academy of Higher Education, Coimbatore, Tamilnadu 641021 India; 5 School of Chemical and Bio Engineering, College of Technology and Built Environment, Addis Ababa University, P.O. Box 1176 Addis Ababa, Ethiopia; 6 Africa Center of Excellence for Water Management, Addis Ababa University, P.O. Box 1176, Addis Ababa, Ethiopia; 7 LUT School of Engineering Sciences, LUT University, Kouvola, Finland

**Keywords:** Life cycle assessment, Waste biomass, Solid acid catalyst, Esterification, Biodiesel production, Chemistry, Energy science and technology, Engineering, Environmental sciences, Materials science

## Abstract

This study presents a combined experimental and life cycle assessment of the production of biodiesel from *Datura stramonium (*DS) seed oil, using a one-pot sulfonated corn-cob solid acid catalyst. The catalyst, synthesized through simultaneous hydrothermal carbonization and sulfonation using sulfuric acid (H₂SO₄), was characterized by Fourier Transform Infrared Spectroscopy (FTIR), showing -SO₃H functionalization; X-ray Diffraction (XRD), indicating an amorphous carbon structure; and Brunauer-Emmett-Teller (BET) surface analysis showed 1.53 m^2^/g surface area, while the catalyst acidity was determined by titration, yielding 1.79 mmol/g. The esterification/transesterification process was optimized to achieve a conversion of 91.35% at 8.75 wt% catalyst loading, 63.2 °C, and 4.8 h(h) reaction time. A cradle-to-gate life cycle assessment (LCA) was performed according to ISO 14040/14044 with Ecoinvent 3.11 APOS system model integrated in OpenLCA 2.5 and the ReCiPe 2016 (H) method for 18 midpoint impact categories. The major environmental hotspots were associated with electricity and solvent consumptions for catalyst sulfonation, oil extraction, and esterification. The measured climate change potential for 17 experimental runs was in the range of 15.40 kg CO₂-eq (minimum impact) to 84.75 kg CO₂-eq (maximum impact), while terrestrial acidification was in the range of 0.129 kg SO₂-eq to 0.239 kg SO₂-eq. In the optimised condition, 15.70 kg CO₂-eq and 0.1306 kg SO₂-eq were obtained, which showed that a proper balance exists between high conversion and low environmental burden, thus confirming the use of corn cob derived acid catalysts for sustainable biodiesel production towards the circular bioeconomy.

**Supplementary Information:**

The online version contains supplementary material available at10.1038/s41598-026-52881-1.

## Introduction

Lignocellulosic biomass residues, such as corncob, are abundant, renewable feedstocks for sustainable materials and biofuels^1^. Corncob is composed of about 40–45% cellulose, 30–35% hemicellulose, and 10–20% lignin, making it a high-carbon precursor for functional carbon materials and heterogeneous catalysts^2,3^. The valorisation of these agricultural residues aligns with global renewable energy strategies, as bioenergy currently supplies approximately 10% of the world’s total energy demand and, under aggressive deployment scenarios, is projected to provide more than one-third by 2050^4,5^. The conversion of corncob into sulfonated solid acid catalysts not only adds value to waste biomass but also supports principles of circular economy and low-carbon energy systems^6,7^. However, despite extensive research on biomass valorisation, many studies remain limited to material synthesis without systematically linking catalyst performance to process efficiency and environmental sustainability, thereby restricting their practical relevance in integrated biofuel systems^8,9^.

In biodiesel production, feedstocks with high free fatty acid (FFA) content require esterification prior to transesterification. Sulfonated carbon catalysts are particularly effective for this purpose, as they catalyse both esterification and transesterification to produce fatty acid methyl esters (FAMEs)^10–12^. Traditionally, various kinds of biomass are carbonized by pyrolysis or hydrothermal treatment; later, this carbon is post-sulfonated with concentrated sulfuric acid or other sulfonation agents. These methods have been time-consuming, energy-intensive, and requiring further chemical additives^13,14^. Moreover, conventional approaches often suffer from limited control over acid site distribution and stability, which can lead to catalyst deactivation and reduced reusability, highlighting a key limitation in translating laboratory-scale catalysts into scalable systems^15–17^.

One-pot hydrothermal process, wherein carbonization and sulfonation occur simultaneously in an aqueous medium with sulfuric acid, has been considered as a sustainable alternative. This method decreases energy consumption and processing time, and it reduces handling of hazardous chemicals by incorporating -SO₃H groups directly during in situ carbon formation^18,19^. These catalysts showed high Brønsted acidity with a high acid site density, which is very crucial for the effective esterification/transesterification of high-FFA oils. Despite such advantages, conventional two-step methods are still widely used, highlighting the need for scalable, environmentally friendly alternatives^20,21^. Nevertheless, the relationship between synthesis conditions, catalyst structure, and long-term catalytic stability in one-pot systems remains insufficiently understood, particularly under liquid-phase esterification conditions, where gradual loss of activity has been frequently observed^22^.

Non-edible oils with high FFA content, such as DS seed oil, are considered promising biodiesel feedstocks due to their favourable fatty acid composition, high oil yield, and non-competition with food crops^23^. Using such low-cost feedstocks enhances economic and energy sustainability while mitigating food-fuel conflicts^24^. However, studies on converting DS seed oil using biomass-derived sulfonated catalysts and evaluating the life-cycle impacts of such processes, particularly when using residues like corncob, remain scarce.

In addition, most existing studies focus on commonly investigated feedstocks such as jatropha, waste cooking oil, and palm oil, while underutilized non-edible oils such as DS have received comparatively limited attention, particularly within integrated catalytic and sustainability assessment frameworks^25–27^.

Integrating corncob-derived one-pot sulfonated catalysts into the biodiesel production pathway for DS seed oil offers dual sustainability benefits: valorising agricultural waste and enabling biodiesel production from a non-edible, high-FFA feedstock^27,28^. However, a critical knowledge gap remains regarding whether one-pot hydrothermal sulfonation can deliver the required porosity, acid-site stability, and catalytic activity for efficient esterification/transesterification, as well as whether the overall process demonstrates sustainability when assessed through a comprehensive LCA. More importantly, previous studies rarely evaluate catalyst synthesis, oil extraction, and biodiesel production as an integrated system, which limits understanding of trade-offs between process efficiency and environmental impacts across the full value chain^9,29^.

In recent years, coupling experimental work with a life cycle assessment (LCA) framework has become increasingly essential to validate the environmental sustainability of biodiesel production systems at an early stage. LCA allows for quantification of greenhouse gas emissions, energy use, and toxicity impacts across feedstock preparation, catalyst synthesis, oil extraction, and fuel conversion^9,30^. Biomass-derived heterogeneous catalysts, in particular, have shown reduced fossil energy demand and ecotoxicity compared to conventional routes^31,32^. However, most existing studies focus on isolated stages of biodiesel production, or partial integration of catalyst synthesis and reaction optimization, without considering the complete process chain from feedstock extraction to fuel conversion within a unified sustainability assessment framework. In particular, few studies simultaneously integrate upstream oil extraction processes, catalyst preparation, reaction optimization using response surface methodology (considering catalyst loading, reaction time, and temperature), and LCA to comprehensively link process performance with environmental impact outcomes. Therefore, a clear gap exists in developing an integrated experimental–environmental framework that simultaneously links feedstock extraction, catalyst synthesis, process optimization, and life cycle impacts within a single system. Based on this gap, the central hypothesis of this study is that a one-pot hydrothermally synthesized corncob-derived sulfonated catalyst, when applied within an optimized reaction system, can achieve high biodiesel conversion efficiency while maintaining improved environmental performance when evaluated across the full production chain.

This study therefore presents a fully integrated experimental and life cycle assessment framework combining Soxhlet extraction of DS seed oil, one-pot hydrothermal synthesis of corncob-derived sulfonated catalyst, and RSM-optimized esterification/transesterification, enabling a comprehensive evaluation of both process efficiency and environmental sustainability.

## Materials and methods

### Materials

Corncob and DS seeds were used as feedstocks for the preparation of a sulfonated solid acid catalyst and oil extraction, respectively. Corncob was collected from Haramaya town (9.40° N, 42.02° E), and the seeds of DS were collected from Bate town (9.72°N, 42.13° E) in January 2025, Oromia Region, Ethiopia with official permission obtained from the relevant local authority. DS identification was carried out by Workisa Bacha and Tesfaye Kassaw. The specimen of the DS was deposited in Haramaya Institute of Technology, Chemical Engineering Department, with the specimen voucher number DS-002/2025 and used strictly following institutional, national, and international guidelines and legislation for experimental research and field studies on wild plants. The raw materials were cleaned thoroughly to remove physical impurities, dried, and size-reduced before the hydrothermal process for catalyst synthesis as well as for oil extraction.

All the chemicals and reagents used in this study were of analytical grade to ensure the validity and accuracy of results obtained from the experiments. The chemicals used were concentrated sulfuric acid (H₂SO₄, 98%), ethanol (C₂H₅OH, 99.9%), methanol (CH₃OH, 99.8%), distilled water, potassium hydroxide (KOH, 85%), potassium iodide (KI, ≥ 99%), sodium hydroxide (NaOH, ≥ 98%), acetic acid (CH₃COOH, ≥ 99.7%), chloroform (CHCl₃, ≥ 99.5%), carbon tetrachloride (CCl₄, ≥ 99%), n-hexane (C₆H₁₄, ≥ 98%), sodium thiosulfate (Na₂S₂O₃, ≥ 99%), and Hanus reagent. The chemicals were all obtained from certified chemical suppliers from Kirkos, in Addis Ababa, Ethiopia and utilized without purification.

### Preparation of catalyst

**Fig. 1:**
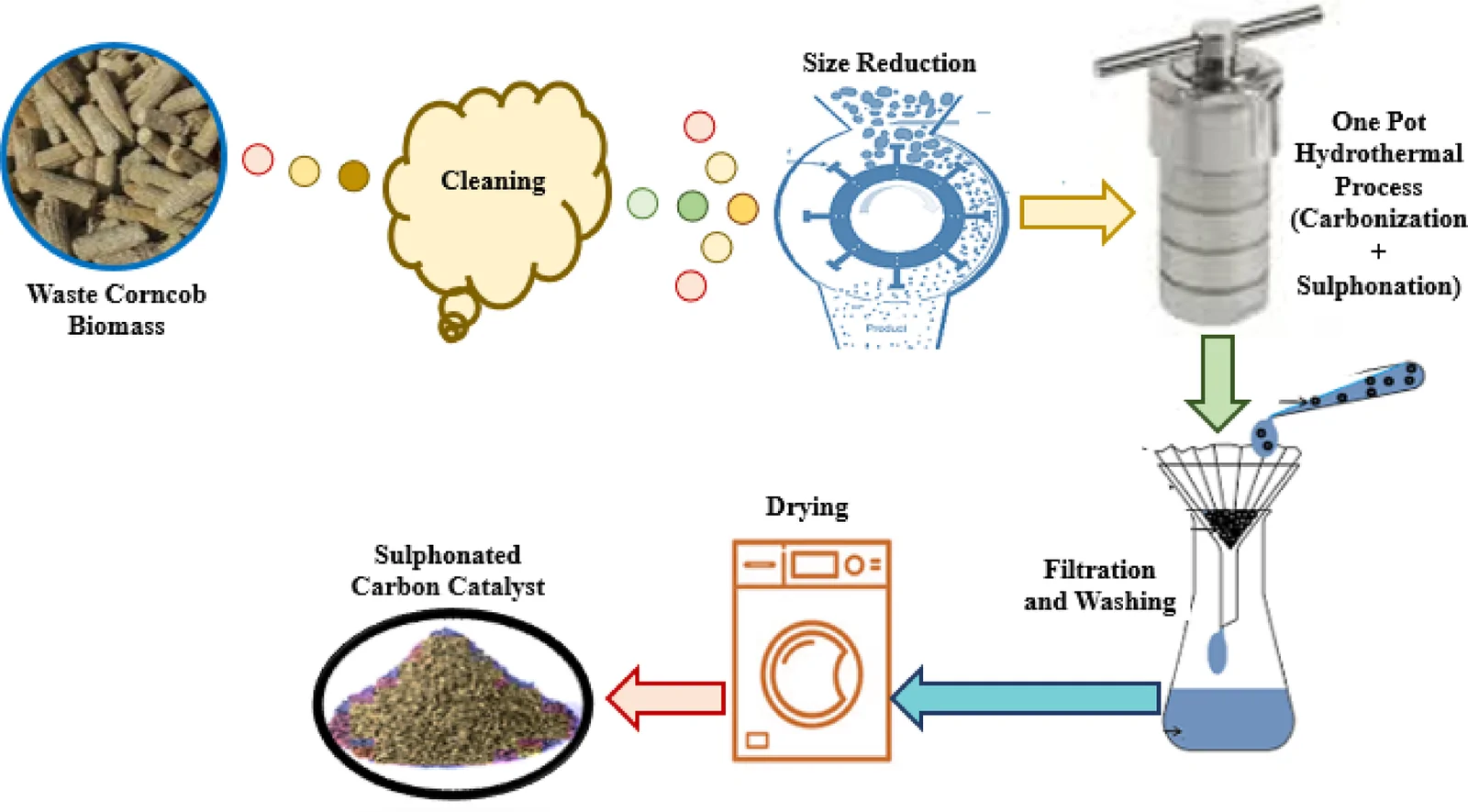
Catalyst preparation process.

The solid acid catalyst was synthesized in a single step via a hydrothermal carbonization-sulfonation process^33^. The catalyst preparation by combining the development of the carbon matrix and the -SO₃H functional group in one step as shown in Fig. 1. Corncob residues were thoroughly washed with deionized water to eliminate any physical impurities, air-dried, and then mechanically ground to obtain a 40-mesh sieve (400 μm) for facilitating increased surface reactivity. The 15 g of ground corncob biomass was mixed with 150 mL of concentrated sulfuric acid at a 1:10 (w/v) solid-to-liquid ratio in a stainless-steel Teflon-lined autoclave and heated at 160 °C for 7 h to promote dehydration, aromatization, and in-situ sulfonation. The produced black solid was cooled slowly, filtered, and washed with hot deionized water to neutral pH before oven drying at 80 °C for 6 h in order to obtain thermally stable sulfonated solid catalyst loaded with -SO₃H, phenolic, and carboxylic groups, which lead to high density acid site and catalytic activity towards esterification/transesterification^34,35^.

### Catalyst characterization

The physicochemical properties of the sulfonated solid acid catalyst were comprehensively characterized by various analytical techniques. Fourier Transform Infrared Spectroscopy (FTIR) was employed to identify surface functional groups with a Bio-Rad Digilab FTS-3500 spectrometer (USA) in the scanning range of 400–4000 cm⁻¹. The presence of sulfonic acid groups (-SO₃H), hydroxyl (-OH), and the other oxygen functions confirmed effective sulfonation and partial carbon surface oxidation. The surface morphology and elemental composition of the catalyst were ascertained by Scanning Electron Microscopy (SEM) and Energy Dispersive X-ray Spectroscopy (EDX) respectively. SEM micrographs revealed the porosity and surface texture of the catalyst while EDX revealed the significant elements like carbon (C), oxygen (O), and sulphur (S) indicating successful sulfonation. X-ray diffraction (XRD) was carried out using a Rigaku D/MAX-B diffractometer (Japan) with Cu Kα radiation (λ = 1.5406 Å) at 40 kV and 30 mA. The patterns were scanned in the range of 2θ values 5° to 50° at a scan rate of 2° per minute. Brunauer-Emmett-Teller (BET) surface area analysis using nitrogen adsorption-desorption isotherms were used to analyse the specific surface area, pore volume, and pore size distribution. Additionally, Thermogravimetric Analysis (TGA) was used to analyse the thermal stability and decomposition behaviour of the catalyst. Acidity of the catalyst was determined by a back titration method using sodium hydroxide (NaOH) as the titrant. For this procedure, 0.1 g of dry catalyst was taken in a blend with 25 mL of 0.05 N NaOH and stirred at room temperature for 24 h. Excess NaOH was titrated with 0.05 N HCl in the presence of phenolphthalein as an indicator. Acid density was obtained from the Eq. 1:

1 $$\:Acid\:density(\mathrm{m}\mathrm{m}\mathrm{o}\mathrm{l}\mathrm{e}/\mathrm{g})\:=\frac{\left({V}_{blank}-{V}_{sample}\right)\times\:N\times\:1000}{W}$$

where, V_blank_ and V_sample_ are blank and sample titration volumes (mL) of HCl, respectively; N is the normality of HCl; and W is the catalyst’s weight in grams. This value provides information about the number of active acidic sites on the catalyst surface, which is especially valuable for determining its activity in catalytic applications such as biodiesel synthesis.

### Oil extraction

Twenty-five-gram milled DS seed powder was extracted in a standard Soxhlet apparatus using analytical-grade n-hexane as extracting solvent. 250 mL hexane volume was used to achieve a solid-to-solvent ratio of 1:10 w/v, and the extraction was carried out at approximately four siphon cycles per hour. The experiment was kept running for 8 h under continuous operation, offering about 32 cycles of siphon to ensure full recovery of the oil^36,37^. The hexane-oil mixture was then concentrated through rotary evaporation under reduced pressure at 40 °C at the end of the extraction to remove the solvent. The crude oil was filtered to purify it and treated with 0.5 M NaOH (pH adjusted to 7) for alkali neutralization to reduce free fatty acids and remove polar impurities. The purified oil was transferred and kept in amber glass bottles at 4 °C for analysis. Oil yield was measured gravimetrically in Eq. 2^38^.

2 $$\:Oil\:Yield\left(\mathrm{\%}\right)\:=\frac{Weight\:of\:Extracted\:Oil\:\left(g\right)}{Weight\:of\:dry\:seed\:sample\left(g\right)}\times\:100$$

### Characterization of oil

#### Density determination

The density of the DS seed oil was determined using a pycnometer based on the American Oil Chemists’ Society (AOCS) Standard Method Cc 10a-25 (2020). First, the pycnometer was calibrated using distilled water at 25 °C and then the oil sample was measured. The density was calculated based on Eq. (3), and the measurements were performed in triplicate, for which the average value was considered.

3 $$\:Density=\frac{Mass\:of\:oil}{Volume\:of\:oil}$$

#### Acid value determination

The acid content of the DS seed oil was determined by titration, according to standard procedure. Approximately 2 g of the oil sample was initially dissolved in 50 mL of ethanol and titrated against 0.1 mol/L KOH using phenolphthalein as indicator. The acid value was subsequently computed from Eq. 4:

4 $$\:\mathrm{A}\mathrm{c}\mathrm{i}\mathrm{d}\:\mathrm{V}\mathrm{a}\mathrm{l}\mathrm{u}\mathrm{e}\:(\mathrm{m}\mathrm{g}\:\mathrm{K}\mathrm{O}\mathrm{H}/\mathrm{g})=\frac{V\times\:N\times\:56.1}{M}$$

In which V is volume of KOH solution used (mL), N is normality of KOH solution (mol/L), and M is the sample of oil weight (g).

#### Saponification value determination

AOCS Official Method was used to carry out saponification value. Approximately 3 g of the test oil sample were weighed accurately. It was mixed with 25 mL of ethanoic potassium hydroxide solution of 0.5 mol/L strength. The sample was refluxed for 30 min so that saponification would be complete. The remaining KOH upon cooling was subsequently titrated with 0.5 mol/L hydrochloric acid (HCl) solution under an indicator, phenolphthalein. The saponification value was determined according to Eq. 5:

5 $$\:Saponification\:Value=\:\frac{\left(B-S\right)\times\:N\times\:56.1}{M}$$

where B = volume of HCl taken by the blank (mL), S = volume of HCl taken by the sample (mL), N = normality of the HCl, and M = sample weight of the oil (g).

#### Determination of free fatty acid content

The free fatty acid (FFA) level of the oil was measured through the normal titration procedure, which gives a direct measure of the degree of hydrolysis and degradation of the oil. Here, the FFA level was measured as a percentage, in oleic acid terms, using Eq. (6). This technique is based on the general premise that oleic acid is the predominant free fatty acid in most vegetable oils and thus serves as an adequate standard for such determinations.

6 $$\:FFA\left(\%\right)=\frac{Acid\:Value}{2}$$

### Esterification/transesterification of oil

The esterification/transesterification of DS seed oil using methanol was carried out in a 250 mL three-neck round-bottom flask equipped with a reflux condenser, using a one-pot hydrothermally synthesized sulfonated corncob catalyst^39^. Preliminary screening experiments were first conducted to identify suitable operating ranges for the key reaction parameters based on conversion trends and practical operating limits.

Based on these initial trials, three independent variables were selected for optimization: catalyst loading (4–10 wt%), reaction temperature (45–65 °C), and reaction time (1–5 h) as shown in Table 1, while the methanol-to-oil molar ratio was fixed at 10:1 to ensure sufficient excess alcohol for esterification/transesterification. These parameter ranges were chosen to avoid mass transfer limitations at low catalyst loadings, minimize thermal degradation at elevated temperatures, and capture the transition from kinetically controlled to near-equilibrium reaction conditions.

A Box-Behnken Design (BBD) was employed within the framework of response surface methodology (RSM) to evaluate the individual and interactive effects of the selected variables on FFA conversion. The design consisted of 17 experimental runs, including 12 factorial runs conducted in triplicate and five replicates at the center point, which were used to estimate experimental error and assess the reproducibility of the system. All experiments were performed in randomized order to minimize systematic bias.

The experimental data were fitted to a second-order polynomial model, and the adequacy of the model was evaluated using analysis of variance (ANOVA), including determination of the coefficient of determination (R^2^), adjusted R^2^, and lack-of-fit tests.

After completion of each reaction, the catalyst was separated by filtration, thoroughly washed with methanol to remove residual reactants and products, and dried in an oven at 80 °C for 9 h prior to reuse in subsequent cycles.

The acid value (AV) of the biodiesel was determined according to SNI 01-3555-1998 and calculated using Eq. (7):

7 $$\:Acid\:Value=\frac{{V}_{KOH}\times\:N\times\:MR}{W}$$

where: V_KOH_ is the volume of KOH (mL), N is the normality of KOH, MR is the molecular weight of KOH (56.11 g/mol), and W is the sample mass in grams.

The percentage conversion of free fatty acids (FFA) was determined using the Eq. 8:

8 $$\:\mathrm{F}\mathrm{F}\mathrm{A}\:\mathrm{C}\mathrm{o}\mathrm{n}\mathrm{v}\mathrm{e}\mathrm{r}\mathrm{s}\mathrm{i}\mathrm{o}\mathrm{n}\:\left(\mathrm{\%}\right)=\frac{{AV}_{i}-{AV}_{t}}{{AV}_{i}}$$

where AV_i_ and AV_t_ are acid values prior to and following esterification/transesterification, respectively.

**Table 1:** Experimental factors for esterification reaction with levels.

Factors	Code	Unit	Coded levels		
			-1	0	1
Catalyst loading	A	Wt%	4	7	10
Temperature	B	°C	45	55	65
Reaction Time	C	h	1	3	5

#### Characterization of biodiesel

Physicochemical properties of the produced biodiesel were thoroughly reported based on international standards. The key fuel properties were defined based on ASTM D6751 and EN 14,214 recommendations. Density was measured by using a digital density meter, and the viscosity was measured at 40 °C using a calibrated viscometer. The cetane number was quantified on a cetane engine and flash point was analysed on a Pensky-Martens closed cup apparatus. Fourier Transform Infrared Spectroscopy (FTIR) was utilized to identify the chemical structure and functional groups of the biodiesel samples, as per methodologies described in previous work^39,40^. The presence, position, and intensity of peaks of absorption were matched with reference spectra to assess the purity of biodiesel and confirm successful transesterification. Gas chromatography-mass spectrometry (GC-MS) identified the FAME composition of the biodiesel. This provided detailed information on the fatty acid profile that is most important in determining combustion behavior, oxidative stability, and overall fuel performance^41,42^. The comprehensive characterization thus confirmed the suitability of the produced biodiesel for use as a renewable fuel.

### Life cycle assessment (LCA)

**Fig. 2:**
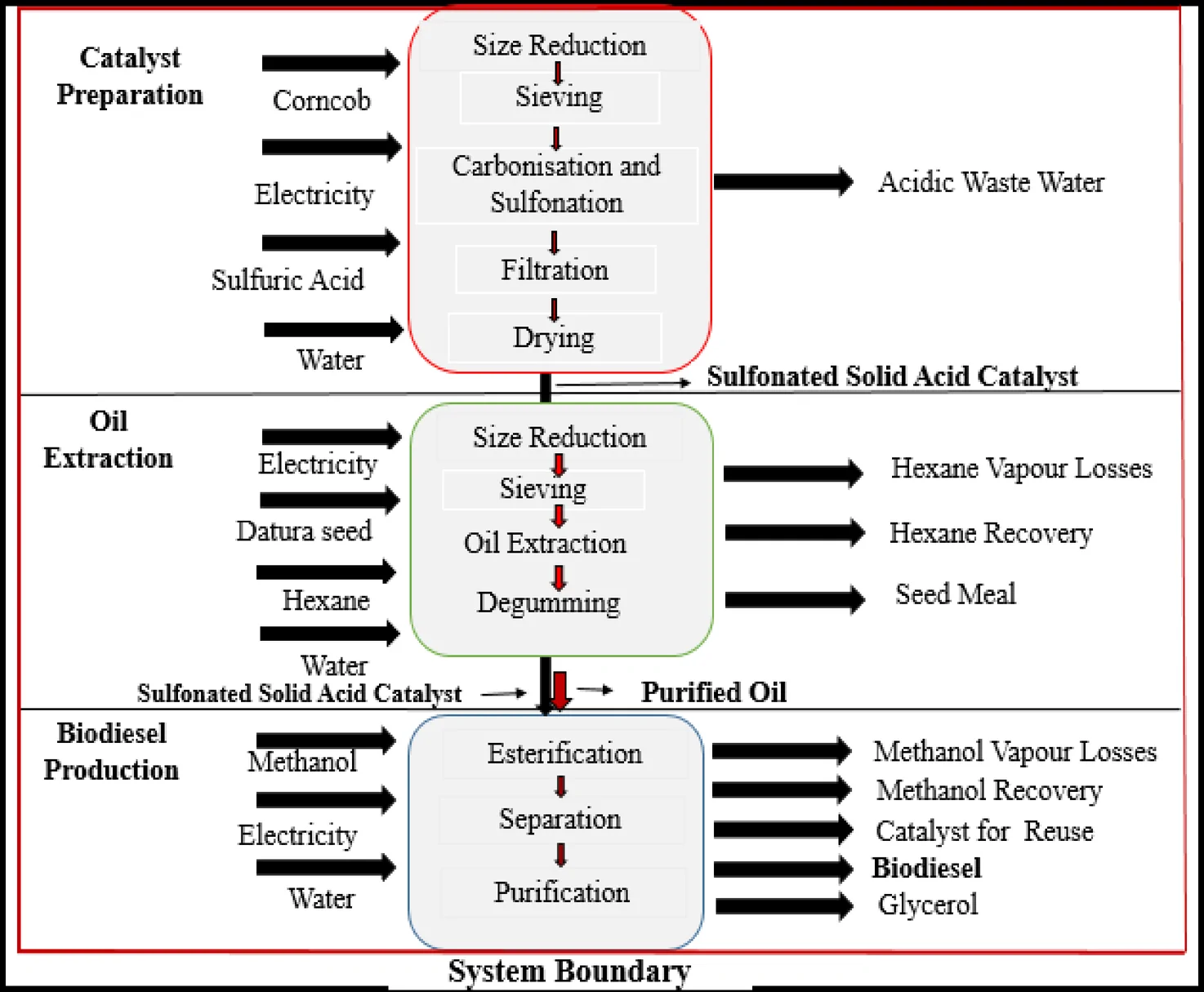
LCA system boundary and interlinked processes.

The life cycle assessment (LCA) of the one-pot sulfonated corn cob solid acid-catalysed biodiesel production from DS seed oil was performed according to ISO 14040 and ISO 14044 standards, covering four steps: goal and scope definition, life cycle inventory, life cycle impact assessment, and interpretation. The primary objective was to assess the environmental burdens of the interlinked process (catalyst synthesis, oil extraction, and esterification) associated with the production of 1 kg of biodiesel as the functional unit. The system boundary was defined as “cradle-to-gate,” encompassing all laboratory-scale operations performed at Haramaya University, Ethiopia. Corn cobs used for catalyst preparation were treated as an agricultural residue remaining after maize harvesting and were therefore assigned zero upstream environmental burdens; in line with ISO 14044 guidance for waste biomass. No cultivation, harvesting, or land use impacts were attributed to the corn cobs, and only their conversion into a sulfonated catalyst was included in the life cycle inventory. Likewise, DS seeds were collected from naturally growing plants within agricultural sites around the university’s research field and were not cultivated as a dedicated feedstock for biodiesel production. Because no fertilizers, irrigation, pesticides, or other agricultural inputs were applied, the seeds were modelled as a biomass by-product with no associated cultivation burdens. Importantly, both the collection of biomasses feedstocks and all experimental work, including catalyst synthesis, oil extraction, and esterification/transesterification, were carried out entirely within the university campus. As a result, transportation requirements, costs, and related emissions were considered negligible and were excluded from the system boundary. Environmental impacts were therefore attributed only to the integrated laboratory scale processes evaluated in this study. A schematic representation showing the LCA system boundary and interlinked processes is presented in Fig. 2.

The first subsystem comprised the synthesis of a sulfonated solid acid catalyst through a one-pot hydrothermal conversion of corn cob biomass. The process used raw corn cob as the feedstock, with concentrated sulfuric acid as the sulfonating agent, deionized water, and electricity supplied for hydrothermal heating. The batch hydrothermal process produced 0.007 kg of sulfonated catalyst, together with acidic wastewater and negligible solid residues. This catalyst batch was not treated as single-use; rather, it was applied across seventeen experimental runs and one optimized run. These seventeen runs represent independent laboratory experiments conducted under different operating conditions and do not correspond to repeated reuse cycles of the same catalyst; therefore, catalyst synthesis impacts were allocated on a mass-based basis according to the catalyst amount used per run, without assuming an extended catalyst operational lifetime. Accordingly, the environmental burdens associated with catalyst synthesis were allocated to individual runs in proportion to the actual catalyst mass consumed, which ranged from 0.000397 to 0.00103 kg per run (0.0009 kg for the optimized condition). This mass-based allocation approach is consistent with ISO 14044 and represents the effective laboratory-scale lifetime of the catalyst. Although catalyst reusability was experimentally demonstrated, catalyst reuse was not explicitly credited in the LCA model, and synthesis impacts were conservatively allocated without accounting for multiple reuse cycles. The oil extraction subsystem extracts the oil from DS seed by using n-hexane as an extraction solvent in the Soxhlet apparatus. Inputs in the oil extraction subsystem include seeds, hexane, and electricity. Outputs included extracted oil, recovered hexane, minor hexane vapour losses, and defatted seed meal. The esterification subsystem involved the conversion of the extracted oil to biodiesel using methanol and synthesized solid acid catalyst under various operating conditions (temperature, catalyst loading, and reaction time). A total of seventeen experimental runs and one optimized run were performed. The inputs were DS seed oil, methanol, catalyst, and electricity, while the outputs of the process consisted of biodiesel, glycerol, recovered and loss methanol, unreacted oil, and reusable spent catalyst.

Life cycle inventory (LCI) was built based on direct experimental data from all 17 and the optimized esterification/transesterification condition experimental runs. Because each run produced a different biodiesel mass, all inputs and emissions were normalized to the 1 kg functional unit by dividing each flow by the biodiesel yield of that run. For clarity, only the laboratory-based inventory of the optimal run is shown in Table 2, while complete input-output datasets for all experimental runs are available in Supplementary Table 1(ST1). Background datasets for upstream processes, such as production of methanol, hexane, sulfuric acid, and electricity generation, were sourced from the Ecoinvent 3.11 APOS system model integrated in OpenLCA 2.5. Co-products (crude glycerol and seed meal) were handled via ISO 14044 compliant mass allocation. Electricity inputs were modeled with the hydroelectricity-dominated grid mix available in the Ecoinvent 3.11 APOS database^43^.

Although each laboratory esterification/transesterification run produced a small amount of biodiesel (0.0085 kg for the optimized condition), all inputs and emissions were normalized to 1 kg biodiesel to comply with ISO 14040/14044 and to enable consistent comparison across experimental conditions. It should be noted that this normalization magnifies laboratory-scale energy and solvent inputs and therefore does not represent industrial-scale biodiesel production. The LCA results are intended for comparative evaluation and hotspot identification among process parameters rather than absolute benchmarking.

**Table 2:** Laboratory-scale mass balance inventory for the optimized experimental run (0.0085 kg biodiesel produced), reported in kg for LCA normalization and subsequently scaled to a 1 kg biodiesel functional unit.

Process	Parameter	Unit	Input	Output
Catalyst synthesis	Corn cob	Kg	0.015	
	H_2_SO_4_	Kg	0.19	
	Distilled water	Kg	0.14	
	Electricity	Kwh	1.52	
	Catalyst	Kg		0.007
	Waste H_2_SO_4_	Kg		0.185
	Waste water	Kg		0.153
Oil extraction	*Datura* seed	Kg	0.025	
	Hexane	Kg	0.165	
	Electricity	Kwh	1.55	
	Oil	Kg		0.00993
	Seed meal	kg		0.01507
	Hexane recovered	kg		0.1648
	Hexane loss	Kg		0.0002
Esterification	Catalyst	Kg	0.0009	
	Oil	Kg	0.00993	
	Methanol	Kg	0.00216	
	Electricity	Kwh	1.156	
	Biodiesel	Kg		0.0085
	Glycerol	Kg		0.00143
	Spent catalyst	Kg		0.0009
	Unconverted oil	Kg		0.000991
	Methanol recovered	Kg		0.0011573
	Methanol loss	Kg		0.0000117

For the life cycle impact assessment (LCIA), ReCiPe 2016 v1.03 midpoint (H) was used to quantify 18 impact categories for the LCIA, namely climate change (kg CO₂-eq), terrestrial acidification (kg SO₂-eq), freshwater and marine ecotoxicity (kg 1,4-DCB-eq), human toxicity (carcinogenic and non-carcinogenic), eutrophication (freshwater and marine, kg P-eq), particulate matter formation (kg PM₂.₅-eq), ozone depletion (kg CFC-11-eq), land use (m²a crop-eq), material and energy resource depletion (kg Cu-eq, kg oil-eq), photochemical oxidant formation (kg NOx-eq), ionizing radiation (kBq Co-60-eq), and water use (m³)^44^. Impact results for all 17 experimental runs and the optimized condition are provided in Supplementary Table S2.

## Results and discussions

### Characterization of catalyst

#### Acid value

Following sulfonation, acid value of the corncob catalyst increased from 0.66 to 1.793 mmol g⁻¹ (-SO₃H contributing 1.103 mmol g⁻¹) shows successful grafting of strong Brønsted sites. While initially the corn cob’s weak -OH and -COOH groups contribute minimal acid activity, incorporation of -SO₃H significantly raises proton-donating power, increasing active-site density and catalytic capability for esterification and transesterification. Similar processes allow for such enhancement: e.g., sulfonated orange peel catalyst with overall acidity of 1.96 mmol g⁻¹ gave 96.5% oleic acid conversion in 60 minutes^45^; likewise, sulfonated camellia oleifera shell biochar containing 2.86 mmol g⁻¹ acid content gave 91% biodiesel under mild conditions and exhibited performance through four cycles of reuse, confirming durability and efficiency^46^. Typically, Yadav et al. indicate that sulfonation reactions always enhance acid site densities, enhancing yields and reaction rates of biomass-derived catalysts^47^. Moreover, high-density SO₃H eucalyptus biochar sulfonated (469 µmol g⁻¹) exhibited 97% FAME yield in oleic acid esterification, indicating the high role of strong acid sites in high-yield conversions^37^.

Consequently, although raw corncob is of low Brønsted acidity, sulfonation transforms it into a good heterogeneous acid catalyst, saturated with -SO₃H sites that improve catalytic performance, especially for high-FFA feedstock like DS seed oil. High density of acid sites combined with demonstrated stability and efficiency in comparable systems makes the C-SO₃H catalyst a viable, eco-friendly alternative for conventional homogeneous or weak solid acids. Table 3 shows the acid value analysis of the corn cob and sulfonated corncob catalyst.

**Table 3:** Acid value analysis of corncob and sulfonated corn cob catalyst.

Materials	Total acid (mmol/g)	-SO_3_H (mmol/g)	-OH (mmol/g)	-COOH (mmol/g)
Corn cob	0.66	-	0.55	0.11
C-SO_3_H	1.7932	1.1032	0.57	0.12

#### FTIR analysis of corn cob, sulfonated corn cob catalyst and recycled catalyst

**Fig. 3:**
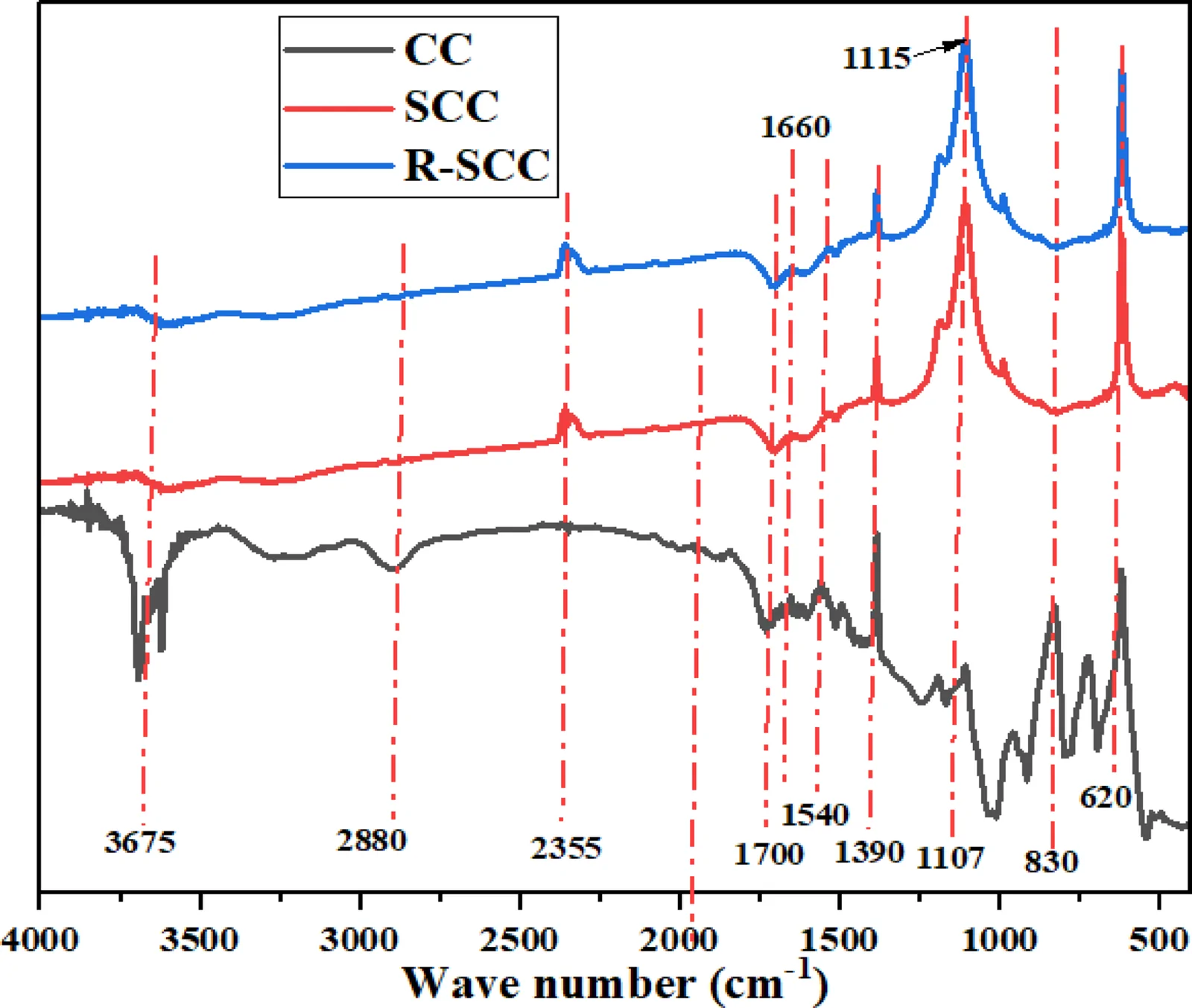
The FTIR spectra of Corn cob, Sulfonated corn cob and Recycled catalyst.

Fourier transform infrared (FTIR) spectroscopy was used to examine the structural changes between the raw corn cob and the sulfonated corn cob catalyst produced through hydrothermal treatment as shown in Fig. 3. The spectrum of the raw corn cob shows a strong and broad band at 3675 cm⁻¹, which corresponds to the stretching vibration of hydroxyl (-OH) groups mainly originating from cellulose, hemicellulose, and lignin in the lignocellulosic structure. After sulfonation, the intensity of this band becomes weaker, indicating that part of the hydroxyl groups participated in the reaction during hydrothermal carbonization and sulfonation. This reduction suggests that these groups served as active sites where chemical modification occurred on the biomass surface during the introduction of sulfonic acid groups^46^. The peak observed at 2880 cm⁻¹ in the raw corn cob sample is associated with aliphatic C–H stretching vibrations from lignocellulosic components. The disappearance of this band in the sulfonated material suggests that some aliphatic chains were altered during hydrothermal treatment, leading to the formation of a more condensed carbon structure. A band at 1700 cm⁻¹ appears in both the raw and sulfonated samples and is assigned to the C=O stretching vibration of carbonyl groups, typically related to carboxylic acids or ester groups derived from lignin and hemicellulose. The presence of this band after sulfonation indicates that oxygen-containing functional groups remain on the catalyst surface, which can contribute to surface acidity and improve interaction with reactant molecules^37,48^. Additional peaks around 1660 cm⁻¹ and 1558 cm⁻¹ correspond to aromatic C=C stretching vibrations, reflecting the presence of partially carbonized aromatic structures that originate mainly from lignin during hydrothermal carbonization. The peaks located at 1490 cm⁻¹, 1390 cm⁻¹, and 1190 cm⁻¹ become weaker after sulfonation, suggesting changes in aromatic structures and C–O functional groups as dehydration and condensation reactions proceed during carbon framework formation. The most noticeable change appears at 1107 cm⁻¹, where a strong peak is observed in the sulfonated catalyst but not in the raw biomass. This band is attributed to the S=O stretching vibration of sulfonic acid (-SO₃H) groups, which confirms that sulfonic acid groups were successfully introduced onto the carbon surface during the sulfonation step. Bands in the 1030–1200 cm⁻¹ region are commonly reported as characteristic signals of -SO₃H groups in biomass-derived sulfonated catalysts and indicate the presence of strong Brønsted acid sites that are responsible for catalytic activity in esterification and biodiesel synthesis reactions^46,49^. In addition, the disappearance of the 830 cm⁻¹ band after sulfonation suggests modification of aromatic C–H bending vibrations due to substitution reactions that occur during the sulfonation process. Taken together, the FTIR results indicate that hydrothermal treatment followed by sulfonation effectively converted raw corn cob into a sulfonated carbon-based solid acid catalyst. The appearance of -SO₃H functional groups together with the remaining oxygen-containing groups and aromatic carbon structure provides stable acidic sites that are suitable for heterogeneous catalytic processes such as esterification and transesterification in biodiesel production^37,46,49–51^.

#### SEM-EDX

**Fig. 4:**
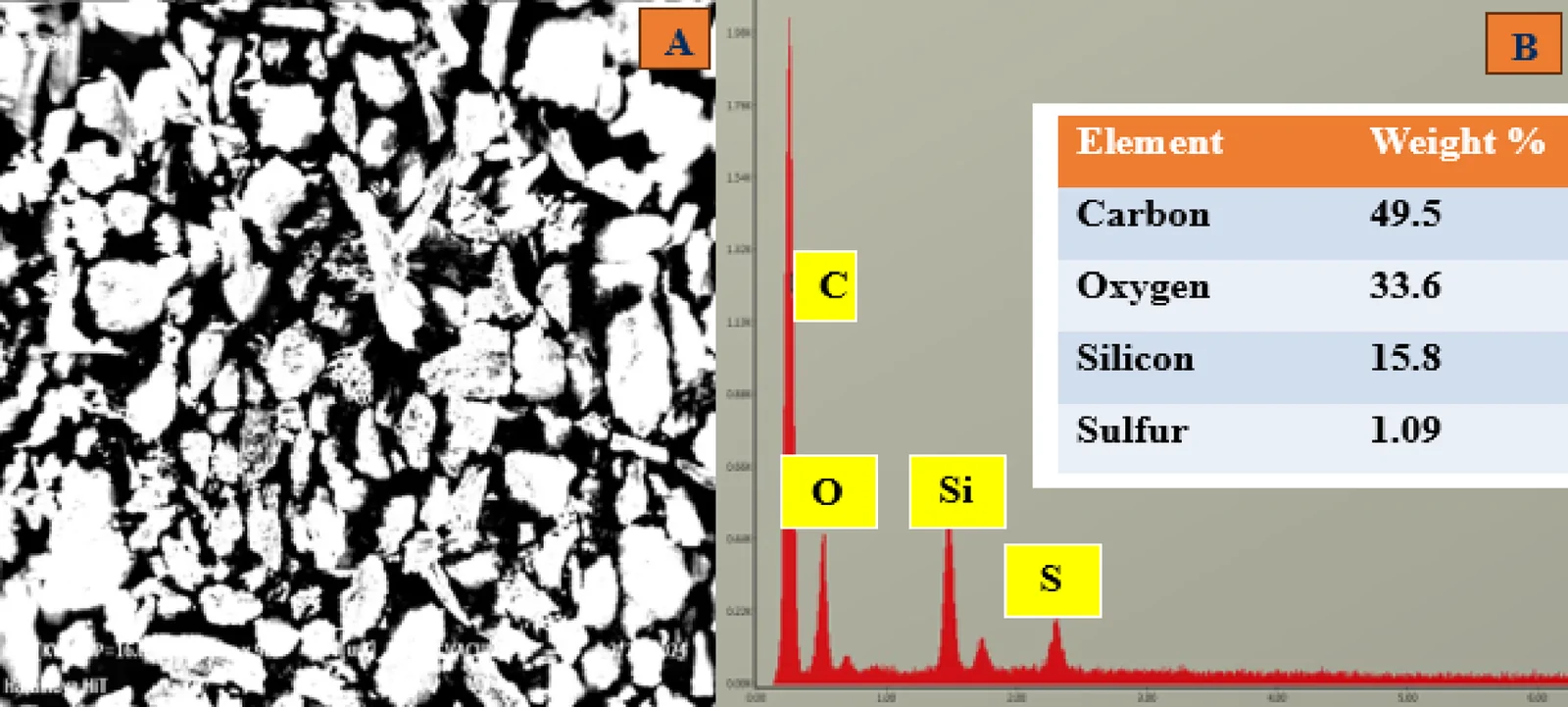
SEM images of sulfonated corncob (**A**) and its EDX spectra (**B**).

SEM micrograph of sulfonated corncob solid acid has a denser, smoother carbon matrix with agglomerated domains, typical of biomass carbons after sulfonation, following partial pore blocking by grafted -SO₃H groups and secondary precipitation of carbon^37^. EDX quantitation (C = 49.5%, O = 33.6%, S = 1.09%, Si = 15.8%) confirms effective functionalization, the characteristic S content (1.09%) is within the range typically range for sulfonated lignocellulosic carbons and sufficient to deliver intense Brønsted acidity to catalyze esterification/transesterification as shown in Fig. 4^37,52^. The Si content arises from biogenic SiO₂ silica present in corncob tissues^53^. This morphology, composition pair, as well as incorporation of S agrees with recent studies on sulfonated biomass-derived catalysts where SEM-EDX verifies sulfur doping and catalytic performance based on acid-site density against retained meso-porosity for diffusion of the substrates^37,52,53^.

#### XRD

**Fig. 5:**
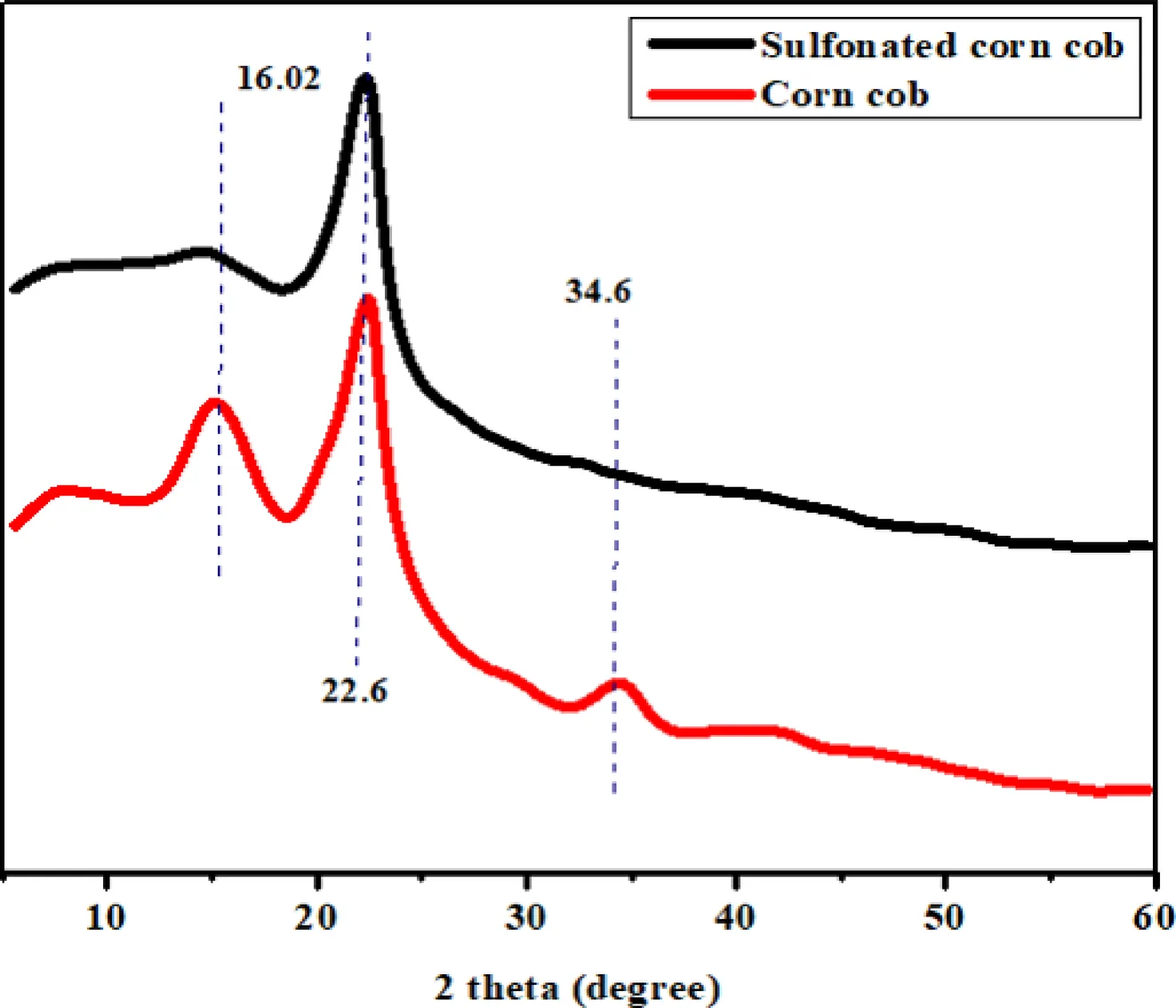
X-ray diffraction (XRD) analysis of corncob and sulfonated corncob.

XRD analysis verifies the structural change from corncob as a biomass-based biopolymer to a carbonaceous amorphous solid upon sulfonation. In the untreated corncob, typical diffraction maxima at 16.0°, 22.6°, and 34.6° are the (110), (200), and (004) planes of the Cellulose-I lattice, which is indicative of the presence of a native semi-crystalline polysaccharide framework^54^. During subsequent sulfonation, these oriented reflections merge into a single broad diffraction band at around 22.6°, typical of the turbostratic (002) band of amorphous carbon and indicate massive disruption of hydrogen-bonded cellulose packing and loss of long-range order caused by functionalization and partial carbonation as shown in Fig. 5^55^.This amorphization phenomenon, transformation of sharp Cellulose-I peaks into a broad halo at 20–23° is well documented for sulfonated cellulose and other sulfonated carbonaceous solids, wherein the introduction of -SO₃H groups, accompanied by dehydration reactions, significantly reduces crystallinity. Corncob biochar and carbons often show a prominent (002) band close to 23° and, in certain instances, a second graphitic (100) band close to 42–44°; however, the lack of the latter in the sulfonated sample indicates an overwhelmingly amorphous, low-graphitic structure^56,57^. Moreover, there are some corncob samples containing crystalline silica (quartz), which yields a sharp peak at 26.6°, but not being detected in the present study implies low siliceous content or concealment of peak by broad amorphous base^58,59^. Collectively, the disappearance of Cellulose-I lattice peaks and the emergence of a single broad (002) peak at 22.6° confirm the successful conversion of corncob to a product that is a sulfonated carbon solid acid amorphous in structure, useful in catalytic applications.

#### TGA

**Fig. 6:**
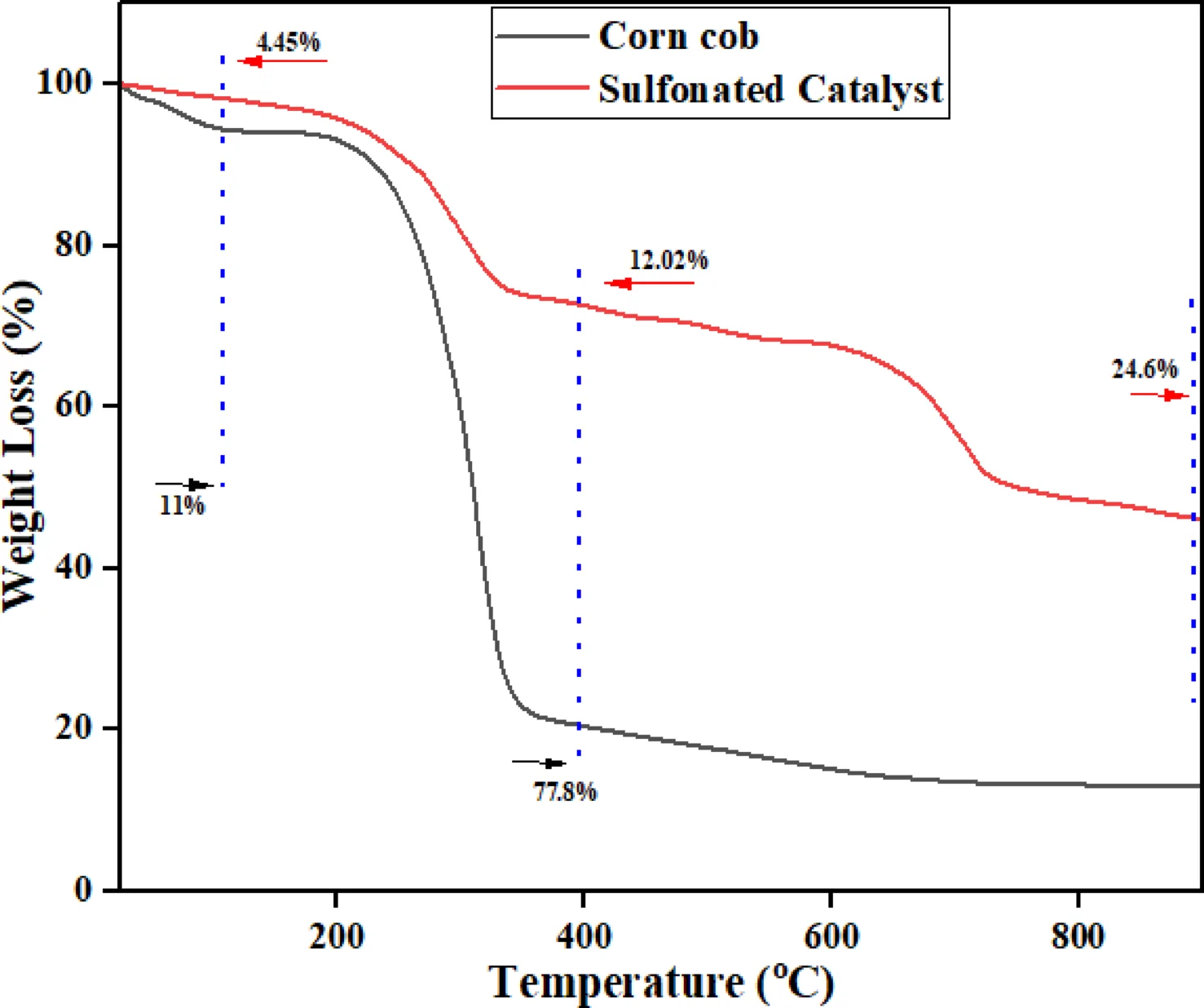
TGA analysis of corn cob and sulfonated catalyst.

Thermal-decomposition profile of raw corncob and sulfonated corncob catalyst was investigated using Thermogravimetric Analysis (TGA), which is displayed in Fig. 6. The TGA profile of non-treated corncob exhibited two clear phases of weight reduction. The first mass reduction of approximately 11% occurred between the temperature range of 35–100 °C because of desorption of physically adsorbed water. The second and most significant weight reduction, about 77.8%, happened in the range of 300–400 °C, that is, thermal degradation of the cellulose and hemicellulose fractions and the partial decomposition of lignin, major constituents of lignocellulosic biomass^56,60^. For comparison, the sulfonated catalyst underwent a modified thermal profile with three degradation stages. The initial stage of 35–100 °C caused an insignificant weight loss of 4.45%, as usual due to evaporation of water. The second stage in the range of 250–400 °C caused a mass loss of 12.02% and resulted from thermal decomposition of the sulfonic acid moieties (-SO₃H) introduced in the sulfonation process. This is in consonance with previous studies indicating that thermal degradation of -SO₃H groups is normally observed within this range^37,61^. The third stage from 780 to 900 °C gave 24.6% loss of mass corresponding to the destruction of the carbonaceous matrix of the catalyst. These results suggest that the –SO₃H groups incorporated into the corncob support matrix exhibit appreciable thermal stability up to approximately 300–350 °C, indicating the suitability of the catalyst for medium-temperature catalytic processes such as esterification/transesterification. The high thermal stability compared to raw corncob is an indication of the effectiveness of the sulfonation process in enhancing the structural strength of the catalyst^47,62^.

#### BET

The surface properties of raw and sulfonated corncob were analysed using BET measurements, and the results are presented in Table 4. Sulfonation significantly altered the textural characteristics, leading to a reduction in surface area from 3.91 to 1.53 m² g⁻¹, which is commonly attributed to pore blockage and structural rearrangement caused by the introduction of -SO₃H and other oxygenated functional groups onto the carbon framework^63^. At the same time, the average pore diameter increased from 12.76 to 23.72 nm, accompanied by a slight increase in pore volume, indicating the development of a more accessible mesoporous structure. This structural evolution is critical because, in liquid-phase esterification/transesterification, catalytic performance is governed not only by total surface area but also by the accessibility and strength of active sites. Similar trends have been reported for one-pot biomass-derived sulfonated catalysts, where relatively low surface areas are compensated by high densities of Brønsted acid sites and improved mass transport. For example, Kumawat and Rokhum reported high biodiesel conversion using a one-pot sulfonated biomass catalyst despite moderate textural properties, attributing performance primarily to accessible -SO₃H groups rather than surface area alone^45^. Likewise, Armylisas et al. demonstrated that single-step sulfonated carbon catalysts can achieve conversions around 90% with limited surface area, emphasizing the dominant role of surface acidity^64^. In comparison, Taslim et al. reported a CaO-rich biomass catalyst with a higher surface area of 9.32 m² g⁻¹ achieving 94.2% conversion; however, this system operates via basic catalysis and employs refined palm olein, which is more uniform and inherently reactive than high-FFA feedstocks^65^. In contrast, the present study uses a sulfonated carbon catalyst for simultaneous esterification and transesterification of DS oil, where larger pore diameter plays a more critical role in facilitating diffusion of bulky triglycerides and free fatty acids. Therefore, despite the low BET surface area, the combination of strong acid site density and enhanced mesoporous accessibility provides sufficient active site availability, enabling effective catalytic performance. This observation is consistent with recent findings that, in biomass-derived solid acid catalysts, catalytic activity correlates more strongly with functional group accessibility and pore structure than with absolute surface area^66,67^.

**Table 4:** BET analysis of Corncob and sulfonated corncob.

Materials	Surface area, SBET(m^2^/g)	Pore volume (cm/g)	Average pore diameter (nm)
Corn cob	3.91	0.078	12.76
Sulfonated corn cob	1.5321	0.088	23.72

### Extraction yield of oil

The oil content of DS seed was calculated to be approximately 41%, a high seed oil content that highlights the plant as a potential feedstock for biodiesel. This yield is particularly noteworthy in comparison to other non-food oil crops such as Jatropha curcas (30–40%) and Azadirachta indica (neem) (25–45%), making DS more competitive than other alternative non edible oils^68–70^. The relatively higher oil content demonstrates that DS would be an economically viable and efficient raw material, especially for low-input and sustainable biodiesel production systems. In addition, DS also possesses other agronomic advantages which make it worthy of being used in biofuel production. As a hardy, water-tolerant crop that can grow on poor or degraded soils with minimal agronomic inputs, DS is a non-competing but sustainable alternative to food oil seeds like soybean, palm, or sunflower^71^. Employing such non-food, underutilized oilseeds reduce the food-versus-fuel debate and is in line with principles of circular bioeconomy and land-use efficiency^36^. Maximization of the oil yield through agronomic practice (e.g., seed pre-treatment and breeding) and enzymatic or ultrasound-assisted oil extraction can additionally enhance the economic feasibility of DS for large-scale biodiesel production^72^. Overall, the obtained extraction yield, combined with the plant’s adaptability and non-edible nature, makes DS a promising candidate for sustainable biodiesel production.

### Characterization of oil

#### Physicochemical analysis of oil

Physicochemical characteristics of DS seed oil promise its prospective employment as an optimal feedstock for the production of biodiesel, as illustrated by Table 5. The oil possessed an acid value of 7.04 mg KOH/g and contained a 3.4% free fatty acid (FFA), so that it proved moderately acidic yet nevertheless fair for processing into biodiesel. A lower free fatty acid (FFA) content and acid value are advantageous, as they minimize soap formation during transesterification and thereby simplify the conversion process. In contrast to this, Jatropha oil reportedly contains acid values of around 15 mg KOH/g, which need additional refining steps before producing biodiesel^73^. Therefore, DS seed oil with comparatively lower acidity is cost-effective because of lesser pre-treatment needs. Although the FFA level of 3.4% is higher than the optimal value of 3% recommended for direct base-catalysed transesterification, it falls within the acceptable range that can be processed by esterification techniques commonly practiced in biodiesel production. Moreover, the density of DS seed oil, measured as 0.903 g/cm³, is well within the acceptable level of biodiesel feedstocks, typically ranging between 0.88 and 0.92 g/cm³^74^. This provides assurance of the compatibility of the oil with standard biodiesel production machinery and engine fuels to be processed and consumed without the need for significant changes. The 231.72 mg KOH/g saponification content also supports the viability of DS seed oil for biodiesel use. The number points to a high proportion of shorter-chain fatty acids, which have been shown to improve cold flow of biodiesel and decrease viscosity and therefore perform better at low temperatures. Similar saponification values have been associated with improved low-temperature performance in other feedstocks utilized for biodiesel^75^.

Generally, the physicochemical analysis shows that DS seed oil possesses some attractive properties, moderate acidity, good density, and optimal saponification value, making it a good non-food and renewable feedstock material for the production of biodiesel.

**Table 5:** Physicochemical analysis of oil.

Properties oil	Unit	Result
Yield	%	41
Acid Value	mg KOH/g	7.04
Free fatty acid	%	3.4
Density	g/mL	0.9633
Saponification value	mg KOH/g	231.72

### Esterification/transesterification reaction of oil

Esterification/transesterification of DS seed oil was conducted under various conditions in order to achieve the highest conversion of free fatty acid (FFA) to esters as shown in Table 6. The methanol-to-oil molar ratio was fixed at 10:1 throughout this study based on preliminary screening experiments, which indicated it as an optimal compromise between conversion efficiency, reaction stability, and downstream separation requirements. Although methanol excess is a key parameter in both esterification and transesterification processes, increasing the molar ratio beyond 10:1 did not lead to a significant improvement in conversion under the studied conditions, while it would have increased methanol recovery demand and associated environmental impacts. Fixing the methanol-to-oil ratio allowed the effects of catalyst loading, reaction temperature, and reaction time to be isolated more clearly, both from a catalytic optimization and a life cycle assessment perspective. This approach also improves the interpretability of the LCA results, as methanol production is a dominant contributor to several impact categories. By maintaining a constant methanol input across all experimental runs, observed environmental differences primarily reflect changes in catalytic efficiency and energy consumption rather than solvent variability.

**Table 6:** Experimental run of esterification/transesterification reaction.

Std	Run	Factor 1	Factor 2	Factor 3	Response 1
		A: Catalyst loading	B: Temperature	C: Reaction Time	Conversion
		Wt%	^o^C	h	%
15	1	7	55	3	77.9
2	2	10	45	3	77.4
11	3	7	45	5	79.1
1	4	4	45	3	68.4
9	5	7	45	1	68.9
3	6	4	65	3	74.3
7	7	4	55	5	72.4
16	8	7	55	3	77.5
6	9	10	55	1	76.1
14	10	7	55	3	78.5
17	11	7	55	3	79.2
10	12	7	65	1	73.5
13	13	7	55	3	78.1
12	14	7	65	5	88.5
5	15	4	55	1	64
4	16	10	65	3	88.1
8	17	10	55	5	89.7

#### Effect of catalyst loading

**Fig. 7:**
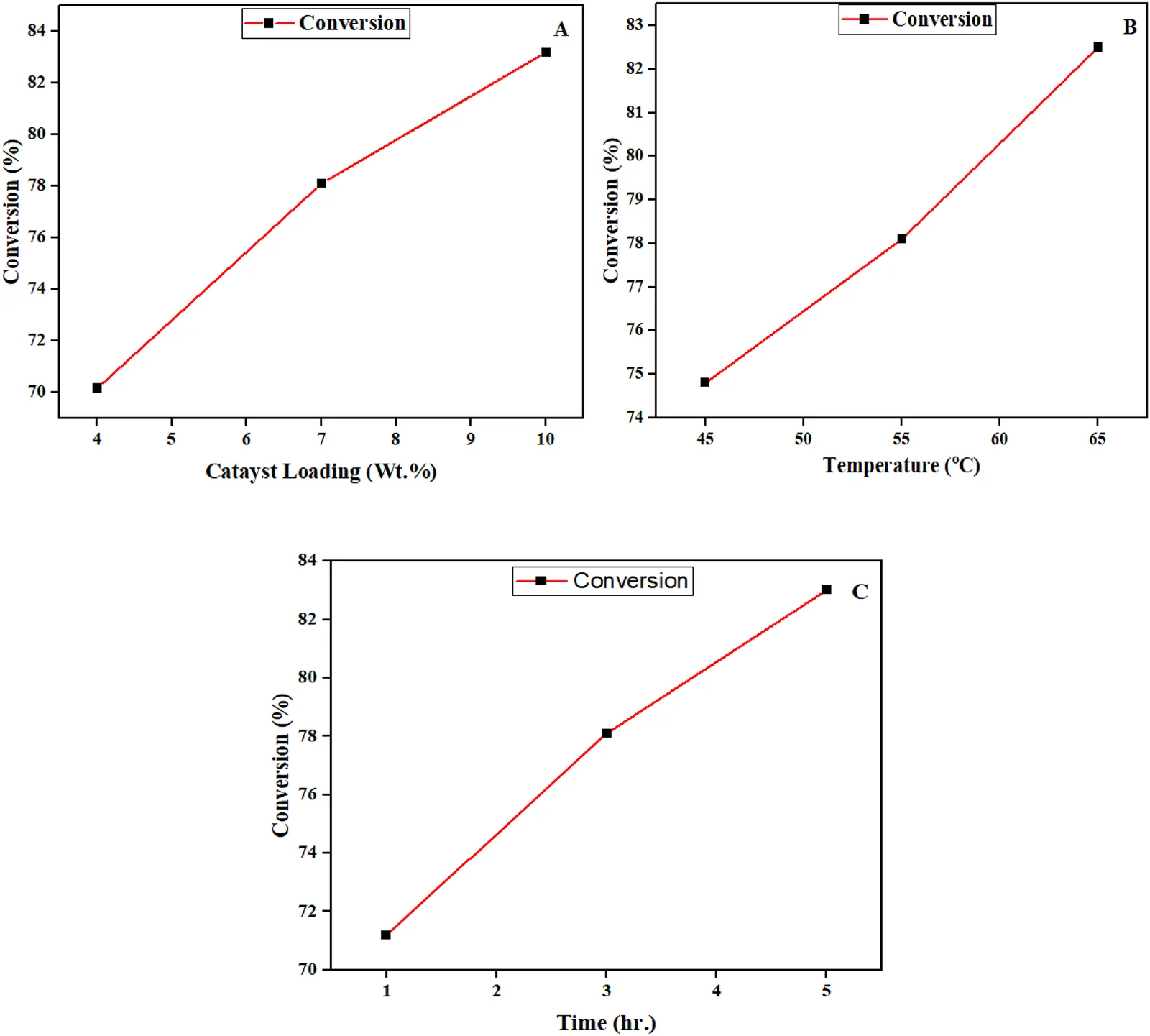
Effect of catalyst loading (**A**), Effect of Temperature (**B**) and, Effect of time (**C**) on conversion.

Catalyst loading was an important factor in biodiesel conversion. Keeping both reaction time and temperature constant at their respective central values (55 °C and 3 h), the increase of catalyst loading from 4% to 10% resulted in a significant increase of conversion from 72.4% to 77.4% as shown in Fig. 7A. It is attributed to an increase of active sites from higher catalyst loading that increases the rate of esterification/transesterification reaction. However, extremely high catalyst concentrations beyond optimized levels may result in diffusion limitations and higher viscosity and therefore lower conversion efficiency^76^. Similar outcomes were reported by Demisu^77^, whose research established that increased concentration of acid catalyst to a certain level, increased esterification/transesterification of non-edible oils.

#### Effect of reaction temperature

Reaction temperature plays a very significant role in esterification/transesterification kinetics. Increasing the temperature from 45 °C to 65 °C while keeping the catalyst loading constant at 7% and reaction time constant at 3 h led to an increase in conversion from 68.9% to 73.5% continuously as shown in Fig. 7B. At elevated temperatures, reactant molecules have enhanced mobility and reduced viscosity with enhanced miscibility of methanol and oil. This improves the collision rate for reactant molecules to enable higher speed conversion. Also, too high a temperature may result in evaporation of methanol and side reactions, which decrease efficiency somewhat^78^. Our results affirm that peak conversion occurs at about 65 °C, consistent with work by Zhang et al.^79^, who demonstrated that higher temperatures up to 65–70 °C facilitate esterification/transesterification without significant thermal degradation of esters.

#### Effect of reaction time

At constant catalyst loading and temperature (7% and 55 °C), reaction time increase from 1 to 5 h raised conversion from 69.1% to 79.1% as shown in Fig. 7C. Longer reaction time allows the reaction to approach more closely to equilibrium and to be completely converted to ester from FFAs. However, beyond a point, the conversion decreases due to back reaction or thermal degradation^47^. Increasing reaction time generally enhances conversion; however, this must be balanced against energy consumption and diminishing returns. In this study, 3 h provided a reasonable compromise, achieving 78.5% conversion, while extending the reaction time to 5 h resulted in only a marginal increase (79.1%), suggesting that the system was approaching equilibrium under the investigated conditions.

#### Interaction of catalyst loading and temperature

**Fig. 8:**
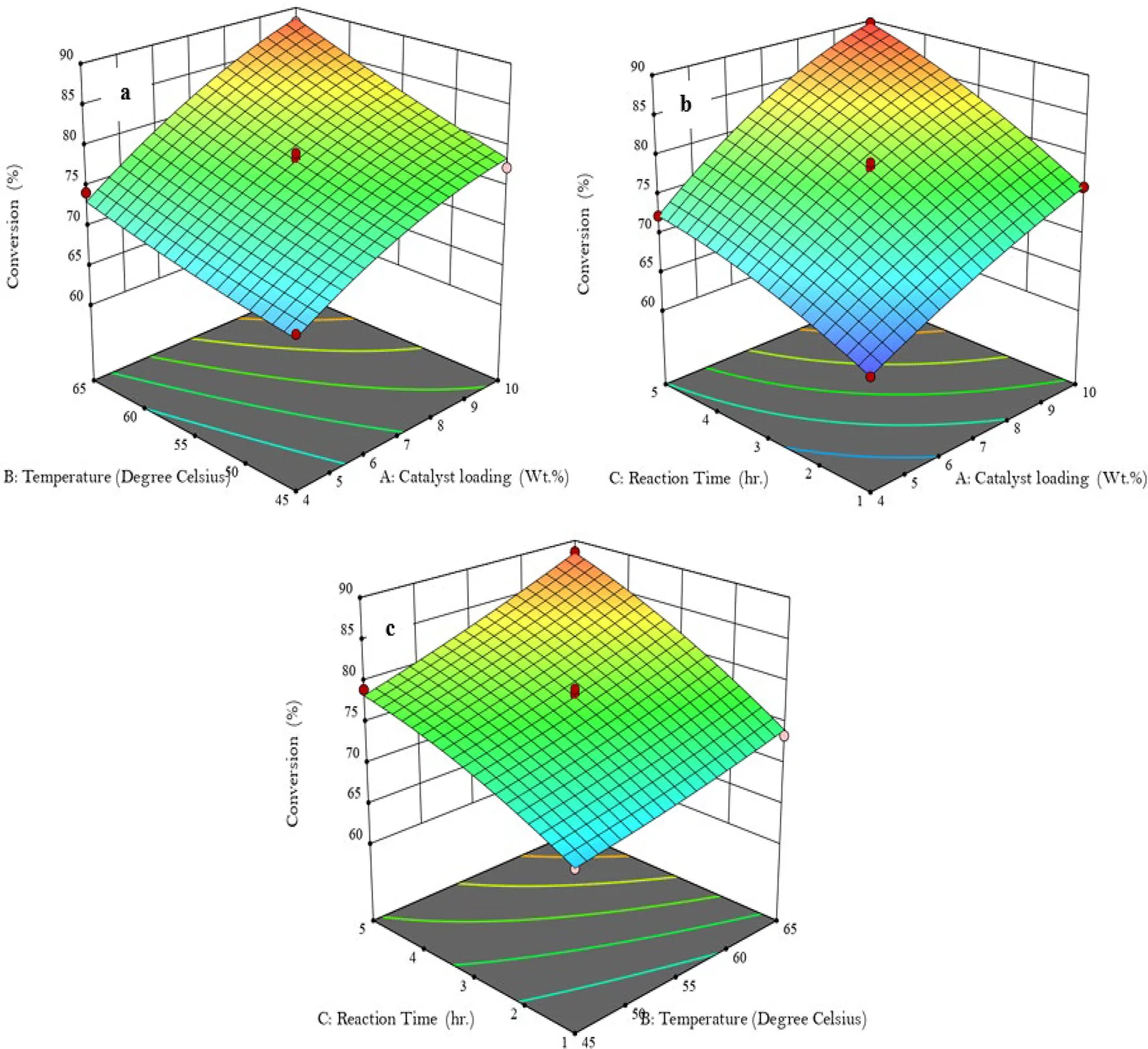
Three-dimensional response surface plots showing the influence of process parameters on conversion: **a** temperature vs. catalyst loading, **b** reaction time vs. catalyst loading, and **c** reaction time vs. temperature.

When reaction time is held constant at 3 h, the interaction between catalyst loading and temperature exerts a pronounced effect on conversion of the DS seed oil esterification/transesterification as shown in Fig. 8a. At low catalyst loading (4 wt%) and low temperature (45 °C) conversion is modest (68%), whereas increasing catalyst loading toward 10 wt% and raising the temperature toward 65 °C yields conversions up to 88–89%. This pattern reflects synergy: increasing catalyst loading boosts the number of active acid sites, while raising temperature enhances kinetics, reduces viscosity, and improves mass transfer. Recent heterogeneous catalyst studies for esterification confirm that conversion increases with both catalyst loading and temperature until a plateau is reached, due to site saturation or equilibrium limitations^61,80^. Thus, in our system the optimum “ridge” in the catalyst loading- temperature plane at C = 3 h appears around 7–10 wt% catalyst loading and 55–65 °C, beyond which further increases afford diminishing returns. From a practical viewpoint, this means that for this one-pot converted solid acid catalyst system, one can trade off modest reductions in temperature with slightly higher catalyst loading (or vice versa) within that optimum region, but pushing both beyond yields little benefit and may incur unnecessary cost or thermal stress.

#### Interaction of catalyst loading and reaction time

Holding temperature at 55 °C, the interplay between catalyst loading and reaction time shows that higher catalyst loading markedly shortens the time needed to reach high conversion, but longer times also amplify conversion especially at higher loading as shown in Fig. 8b. For example, at 4 wt% catalyst and 1 h reaction time conversion is 64%, increasing to 72% when time is extended to 5 h; at 10 wt% loading and 1 h time conversion is 76%, and this climbs to 89.7% when time is 5 h. This indicates that catalyst loading and reaction time interact positively: more loading drives faster kinetics and shorter times; conversely longer time allows fuller utilization of active sites especially when catalyst loading is ample. These dynamics mirror findings in esterification/transesterification optimization studies where both catalyst concentration and time were significant interactions^61^. Therefore, in our catalyst loading- reaction time plane at temperature = 55 °C the surface rises steeply with increasing loading at shorter times, and flattens as time increases beyond 3–5 h at higher loading. This suggests that if short reaction time is required, investing in higher catalyst loading is effective; but if catalyst cost is a constraint, extending reaction period may partially compensate, though beyond a threshold time the improvements are limited.

#### Interaction of temperature and reaction time

With catalyst loading fixed at the centre value of 7 wt%, the interactive effect between temperature and reaction time becomes evident: conversion increases with both parameters, but the effect of reaction time is more sensitive at moderate temperatures, and high temperature amplifies the benefit of longer time as shown in Fig. 8c. At 45 °C and 3 h reaction time conversion is 77.9%; raising time to 5 h yields 79.1%. By contrast at 65 °C and 1 h time the conversion is 73.5%, but extending to 5 h gives 88.5%. Thus, higher temperature alone does not guarantee high conversion unless sufficient time is provided; likewise, longer time at lower temperature gives only modest gains. This corresponds with principles in heterogeneous esterification where higher temperature reduces viscosity, enhances mass transfer and rate, but full conversion still depends on adequate contact time and equilibrium attainment^81^. Accordingly, in the temperature-reaction time plane at catalyst loading of 7 wt%, the surface shows low conversion in the region of low temperature/low time, but steep increases when both temperature and time are elevated. The observed steep gradient indicates synergy: raising one parameter increases the sensitivity to the other. From an operational viewpoint, for a fixed catalyst loading, one may opt for moderate temperature if time is abundant, or increase temperature when shorter times are required, but both must be balanced.

### Statistical evaluation and predictive modeling of esterification/transesterification

Statistical analysis confirmed that the quadratic model for DS seed oil esterification/transesterification using the sulfonated corncob catalyst is predictive and statistically significant. ANOVA results at Table 7 show that the model is significant with F-value = 64.90 and p-value < 0.0001, so the probability of obtaining such results by random noise is less than 0.01%. Key variables, catalyst loading (A), temperature (B), and reaction time (C) are highly significant, with very low p-values (< 0.0001) and high F-values (257.98, 88.65, and 210.92 respectively), indicating their very strong impact on biodiesel conversion. Although interaction terms (AB, AC, BC) were statistically not significant at the 95% confidence level (*p* > 0.05), they are included for the hierarchical model structure. Surprisingly, the quadratic term for catalyst loading (A²) was significant (*p* = 0.0264), confirming a non-linear effect, while B² and C² were not. The non-fit of the model was not significant statistically (*p* = 0.0575), and the model fits well with the experimental data. Fit statistics as shown in Table 8, evidently support the above conclusion, and the coefficient of determination (R²) = 0.9882, adjusted R² = 0.9729, and predicted R² = 0.8414, and the differences among these are < 0.2, which shows the predictability of the model is good. The Adequate Precision value of 28.20 is significantly greater than the threshold requirement of 4 and indicates an excellent signal-to-noise ratio. These results are consistent with previous studies emphasizing the significance of catalyst concentration, temperature, and reaction time in esterification/trasesterification efficiency in the production of biodiesel from high FFA feedstocks^82,83^. The ultimate regression model, in coded factor terms, is as follows in Eq. 9:

9 $$\:Conversion\:=78.24+6.52\mathrm{A}+3.82\mathrm{B}+5.90\mathrm{C}+1.20\mathrm{A}\mathrm{B}+1.30\mathrm{A}\mathrm{C}+1.20\mathrm{B}\mathrm{C}-1.57{A}^{2}+0.38{B}^{2}-1.12{C}^{2}$$

where A, B, and C are coded levels of catalyst loading, temperature, and reaction time, respectively. The model in general confirms that catalyst loading has the highest linear influence on conversion, followed by time and temperature, and is amenable to be used reliably for optimization and scale-up of biodiesel production from non-edible oils such as DS seed.

**Table 7:** ANOVA for quadratic model.

Source	Sum of Squares	DF.	Mean Square	F-value	*p*-value	
Model	771.12	9	85.68	64.90	< 0.0001	Significant
A-Catalyst loading	340.60	1	340.60	257.98	< 0.0001	
B-Temperature	117.04	1	117.04	88.65	< 0.0001	
C-Reaction Time	278.48	1	278.48	210.92	< 0.0001	
AB	5.76	1	5.76	4.36	0.0751	
AC	6.76	1	6.76	5.12	0.0581	
BC	5.76	1	5.76	4.36	0.0751	
A²	10.38	1	10.38	7.86	0.0264	
B²	0.6080	1	0.6080	0.4605	0.5192	
C²	5.28	1	5.28	4.00	0.0856	
Residual	9.24	7	1.32			
Lack of Fit	7.57	3	2.52	6.04	0.0575	Not significant
Pure Error	1.67	4	0.4180			
Cor Total	780.36	16				

**Table 8:** Model fit statistics.

Statistic	Value	Interpretation
R²	0.9882	Indicates that 98.82% of the variation in conversion is explained by the model
Adjusted R²	0.9729	Adjusted for the number of predictors; still very high
Predicted R²	0.8414	High prediction reliability; difference < 0.2 from Adjusted R²
Standard Deviation	1.15	Low variability among residuals
Coefficient of Variation (C.V.%)	1.49	Low CV indicates high precision and repeatability
Adeq. Precision	28.20	> 4 is considered adequate; indicates a strong signal-to-noise ratio

**Fig. 9:**
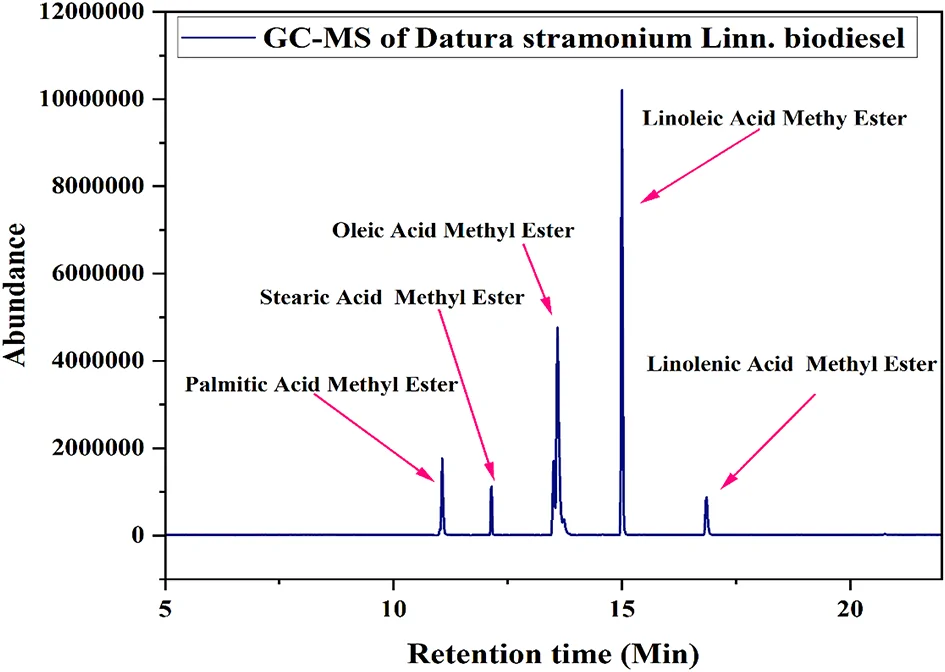
Predicted vs. actual value.

Comparison of actual and predicted biodiesel conversion values indicates that the quadratic regression model built has good fit and high prediction accuracy in the experimental design space as shown in Fig. 9. Actual values closely follow predicted values for all 17 runs with small residuals and no systematic bias. For instance, minimum actual conversion (64.00%) was correctly predicted with a very high rating (64.43%), and similarly maximum experimental yield (89.70%) was correctly predicted as 89.28%, which reflects good performance of the model at both extremes. This close correlation, coupled with a high R² value (0.9882) and low standard deviation (1.15), reflects strong model reliability in predicting outcomes. These results are consistent with earlier modeling experiments in esterification where good predicted vs. actual plots are used as main measures of model adequacy and lack of bias^84,85^. The minor discrepancy between predicted and actual responses indicates that experimental noise and residual variation were adequately captured by the model, which is essential for reliable simulation and process scale-up.

### Optimization and model validation

At the optimization stage, maximum biodiesel conversion was targeted while minimizing catalyst loading, with temperature and reaction time maintained within practically relevant ranges. Numerical optimization based on the desirability function identified an optimal point at 8.75 wt% catalyst loading, 63.2 °C, and 4.82 h, with a predicted conversion of 91.52% and a desirability value of 1.000. Experimental validation under these conditions resulted in a conversion of 91.35%, showing excellent agreement with the predicted value and confirming the robustness and predictive capability of the response surface methodology (RSM) model.

When benchmarked against recent studies, higher biodiesel conversions (95–99%) are frequently reported under shorter reaction times and lower catalyst dosages. For example, Tambun et al. achieved a conversion of 97.86% within 60 min using only 1 wt% KOH under vacuum conditions^86^, while Burmana et al. demonstrated stable performance of Amberlyst CM-4 over multiple reuse cycles^87^, and Burmana et al. reported conversions up to 98.5% using lauric acid as a model feedstock^88^. Similarly, Tambun et al. obtained conversions exceeding 96% under vacuum-assisted esterification conditions^89^. However, direct comparison is constrained by fundamental differences in feedstock composition, catalyst characteristics, and operating conditions. The studies predominantly employ refined or model substrates such as palm olein or lauric acid, which are relatively homogeneous and more reactive. In contrast, DS oil is a high-FFA, non-edible feedstock with a more complex composition, requiring simultaneous esterification and transesterification. This increases mass transfer resistance and slows reaction kinetics, thereby limiting achievable conversion under comparable conditions. In addition, differences in catalyst characteristics further explain the variation in performance. Homogeneous catalysts and commercial ion-exchange resins provide highly accessible active sites, enabling rapid reaction rates^86,87^, whereas the one-pot hydrothermally synthesized sulfonated corncob catalyst used in this study is a biomass-derived solid acid with less uniform distribution and accessibility of -SO_3_H groups. This structural heterogeneity can reduce effective active site availability, particularly in viscous, high-FFA systems. Furthermore, the referenced studies employ process intensification strategies, especially vacuum-assisted operation, which enhances methanol evaporation, improves mass transfer, and shifts reaction equilibrium toward ester formation^86,89^, thereby significantly reducing reaction time and increasing conversion. In contrast, the present work was conducted under conventional reflux conditions without such intensification, which partly explains the longer reaction time (4.8 h) and the observed conversion level.

From a broader perspective, the performance achieved in this study aligns well with reported trends for one-pot biomass-derived sulfonated catalysts, which typically deliver biodiesel conversions in the range of approximately 90–98%, depending on feedstock complexity and catalyst structure. For example, a waste-derived sulfonated carbon catalyst prepared via a single-step process has been reported to achieve around 92% conversion under comparable conditions^64^, while higher conversions (> 95%) are generally associated with model feedstocks such as oleic acid or with catalysts exhibiting more uniform structure and improved acid site accessibility^45,90^. In this context, the slightly lower conversion observed in the present work can be understood in relation to the use of a complex, high-FFA feedstock and a biomass-derived catalyst with inherently heterogeneous active site distribution. Nevertheless, the system demonstrates a balanced and practically relevant performance by combining moderate reaction conditions, a low-cost and sustainable catalyst derived from agricultural waste, and a simplified one-pot synthesis route. More importantly, this study advances beyond conventional approaches by integrating catalyst synthesis, oil extraction, process optimization, and life cycle assessment within a unified framework, thereby enabling a more comprehensive evaluation of both process efficiency and environmental sustainability, which is often overlooked in studies focused solely on catalytic performance.

Overall, the results confirm that the developed catalyst system is capable of achieving high conversion (> 90%) from a complex, non-edible feedstock while maintaining process simplicity and sustainability, which are critical considerations for practical biodiesel production.

### Characterization of biodiesel

#### Physicochemical analysis of biodiesel

Biodiesel should undergo physicochemical characterization to evaluate its quality. As shown in Table 9, the physicochemical properties of the biodiesel indicate its potential as a viable fuel. The density of the biodiesel found in this study was 0.82 g/cm³, which is in good agreement with the accepted biodiesel standards, which normally fall between 0.86 and 0.88 g/cm³^91^. Because of its low density, it may be able to combine well with traditional diesel fuels, increasing fuel efficiency and possibly lowering emissions during burning. The measured kinematic viscosity of 3.8 mm²/s falls between the ideal range of 3.5 to 5.0 mm²/s for biodiesel. In diesel engines, this viscosity guarantees efficient fuel atomization and injection, which enhances combustion properties. Furthermore, it was determined that the biodiesel’s cetane number was 51, which is the bare minimum needed for diesel engines to operate at their best during combustion. Better ignition quality is indicated by a higher cetane number, which promotes more effective combustion and lower emissions of unburned hydrocarbons^92^. Additionally, it was found that the biodiesel’s flash point was 56 °C, which is far higher than that of conventional diesel fuels. This higher flash point denotes improved handling and storage safety, reducing the possibility of fire threats.

**Table 9:** Physicochemical analysis of biodiesel.

Properties of biodiesel	Unit	Result
Kinematic Viscosity	mm²/s	3.8
Cetane number		53.4
Density	g/mL	0.902
Flash point	^o^C	56

#### Comparative FTIR analysis of oil and biodiesel

**Fig. 10:**
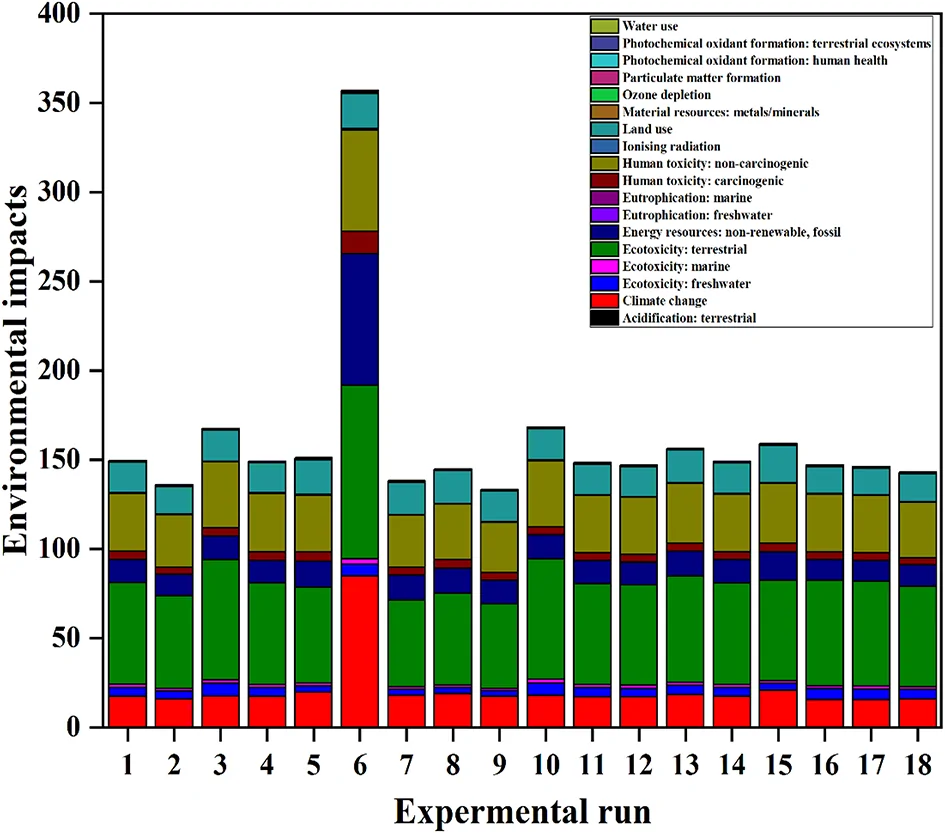
Comparative FTIR analysis of *Datura stramonium* seed oil and its biodiesel.

In FTIR analysis of DS seed oil, the weak band at 3453 cm⁻¹ (free O-H due to moisture/traces of glycerol) typically vanishes after transesterification and washing in line with loss of glyceridic impurities (and reduced hydrogen-bonded OH) in the biodiesel spectrum; concurrently, the cis-olefinic =C-H stretch at 3008 cm⁻¹ is the same intensity in oil and biodiesel, signifying preservation of unsaturation (mainly oleic/linoleic chains) as shown in Fig. 10^27,93^. The intense methylene bands at 2922 and 2853 cm⁻¹ remain in both compounds since they reflect the long aliphatic chains found in triglycerides and their methyl esters^93^. The most indicative change is the carbonyl area, the ester C=O band at 1744 cm⁻¹ in triglyceride oil but tending to sharpen and intensify and slightly shift to higher wavenumber in the biodiesel, as FAMEs are in a more homogenic carbonyl environment than mixed acylglycerols^94,95^. Consequently, C-O related features become more intense in the biodiesel, the band at 1238 cm⁻¹ (C-O stretching of esters) and the 1169 –1060 cm⁻¹ fingerprint region (C-O-C/C-O stretching) tend to give rise to sharper, higher-contrast peaks in FAMEs compared to the parent oil, and are regularly used as spectral indicators of successful conversion^93,95^. Finally, the long-chain (CH₂)ₙ rocking band at 721 cm⁻¹ appears in both spectra with equivalent position and intensity, confirming maintenance of extended aliphatic packing after transesterification^27,93^. Together, these trends, reduced O-H, retained = C-H at 3008 cm⁻¹, strong CH₂ stretches, more defined/stronger 1740–1745 cm⁻¹ carbonyl, and stronger C-O/C-O-C bands are in agreement with successful conversion of DS oil to its fatty-acid methyl esters.

#### GC-MS analysis of biodiesel

**Fig. 11:**
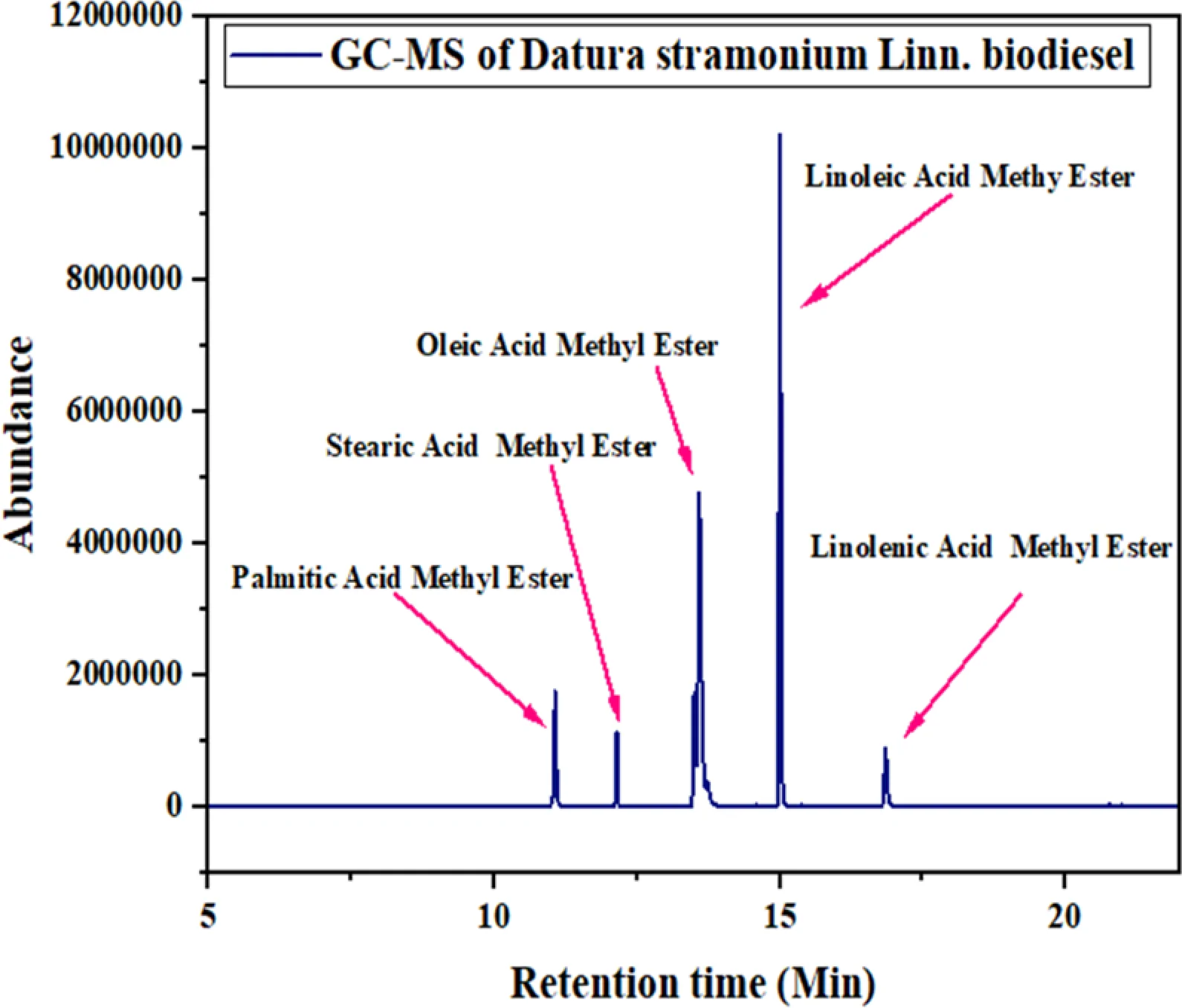
GC-MS analysis of biodiesel.

GC-MS analysis of DS seed oil biodiesel revealed a composition dominated by C18 unsaturated methyl esters, with methyl linoleate (C18:2) and methyl oleate (C18:1) accounting for 47.4% and 32.8% of the total fatty acid methyl esters (FAMEs), respectively as shown in Fig. 11. There was a small contribution from methyl linolenate (C18:3, 2.6%), while the saturated fraction was comprised predominantly of methyl palmitate (C16:0, 10.9%) and methyl stearate (C18:0, 6.3%), for a total saturation of approximately 17.2%. This very unsaturated composition is consistent with recent study on DS seed oil, in which linoleic and oleic acids were also the major constituents^27^.

On the aspect of fuel performance, the high proportion of unsaturated, specifically oleic and linoleic methyl esters (80.2%), will be expected to have good low-temperature operation. High concentrations of C18:1 and C18:2 have been strongly linked to reduced pour point (PP) and cold filter plugging point (CFPP) due to the lower crystallization tendency of unsaturated chains compared to long-chain saturates^96,97^. But the same enrichment in polyunsaturated methyl esters (C18:2 + C18:3 = 50.0%) poses an oxidative stability problem, as bis-allylic positions in linoleic and linolenic esters are highly prone to autoxidation by hydrogen abstraction and peroxyl radical propagation^98^. The trade-off between enhanced cold flow properties and reduced oxidative stability is well documented in the biodiesel literature, with common mitigation strategies including antioxidant addition, metal deactivation, and blending with less unsaturated feedstocks^27,99^.

Importantly, the linolenic acid methyl ester content (2.6%) in the biodiesel is well below the maximum limit of 12% (m/m) specified by EN 14,214 biodiesel standard due to oxidative stability concerns, thereby satisfying a key international quality requirement. The observed FAME profile also indicates a cetane number (CN) within acceptable biodiesel ranges, based on the balance between monounsaturated and saturated esters. However, the relatively high degree of polyunsaturation is expected to slightly reduce the CN compared to more saturated biodiesels^97^. Overall, the composition of the produced biodiesel, which is rich in oleic and linoleic esters with low saturated content and moderate linolenic levels, suggests good cold-flow properties. As a result, the DS biodiesel meets most key fuel quality requirements and can serve as a viable diesel substitute, provided that proper oxidative stability measures are applied during storage and distribution.

### Catalyst reusability

**Fig. 12:**
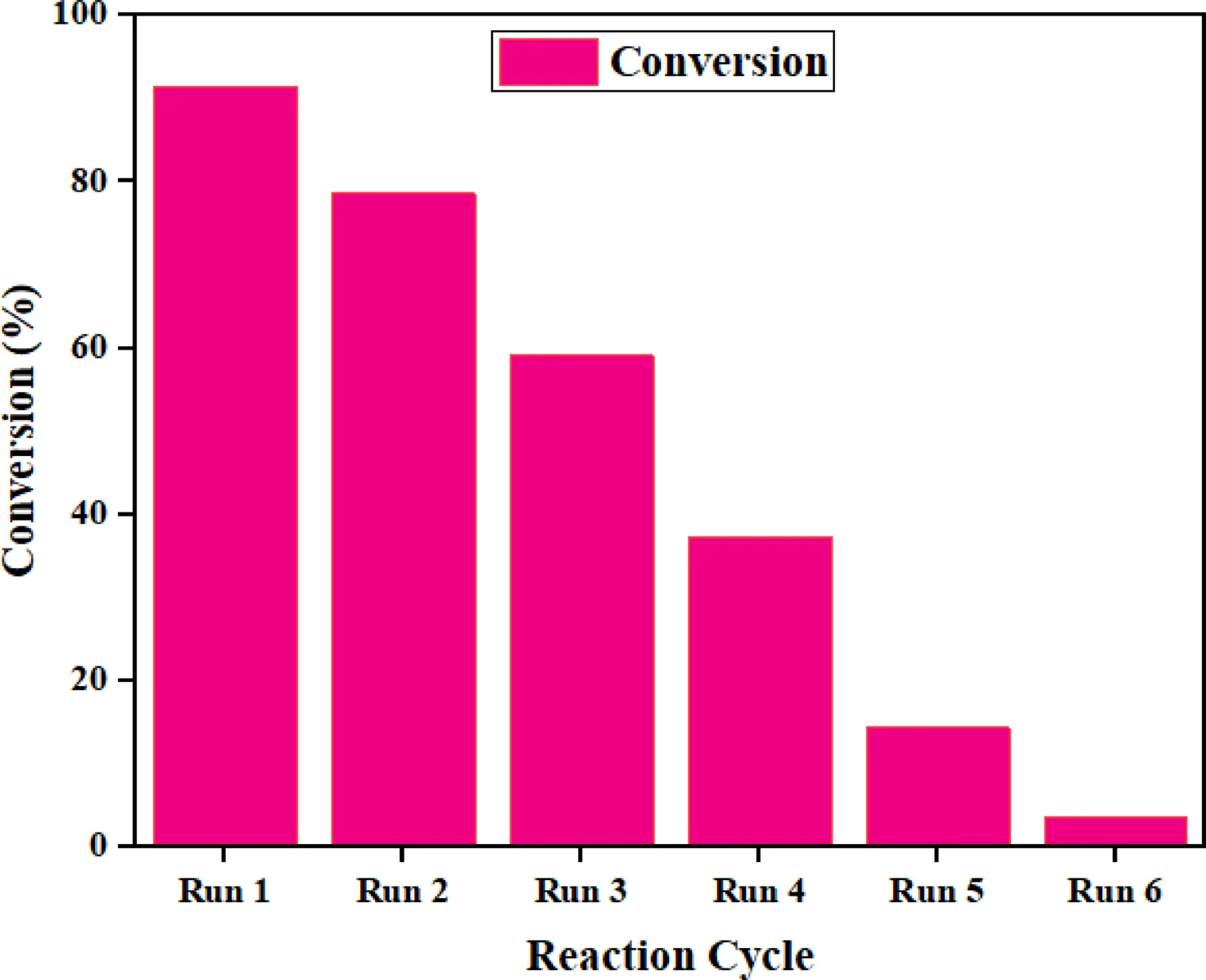
Catalyst reusability.

The reusability of the prepared catalyst was evaluated over six consecutive esterification/transesterification cycles under the optimized conditions of 8.76 wt% catalyst loading, a reaction temperature of 63.2 °C, and a reaction time of 4.8 h. After each cycle, the catalyst was recovered by decantation and filtration, followed by washing with methanol to remove residual reactants and products. As illustrated in Fig. 12, the catalytic activity progressively declined with increasing reuse cycles. The conversion decreased from 91.52% in the initial run to 3.6% in the sixth cycle, indicating a significant loss in catalytic performance.

A noticeable reduction in conversion was observed after the first reuse cycle (78.6%), corresponding to a decrease of approximately 14.1%. This initial decline may be associated with partial leaching of surface acidic functional groups, particularly -SO_3_H moieties, which can detach during liquid-phase reactions. Similar behaviour has been reported for sulfonated carbon-based catalysts, where the gradual loss of active sites contributes to reduced catalytic activity over repeated use^22^.

Further decreases in conversion were observed in subsequent cycles, reaching 59.1% by the third cycle. This trend suggests progressive catalyst deactivation, which may arise from a combination of active site loss and surface fouling. FTIR analysis of the reused catalyst (Fig. 3) shows that the characteristic S = O stretching band associated with -SO_3_H groups remains present, with a slight shift from 1107 cm⁻¹ to approximately 1115 cm⁻¹. Such a shift may reflect changes in the local chemical environment of the functional groups during repeated reaction cycles^22,64^. The carbonyl band near 1700 cm⁻¹ remains visible in both fresh and reused catalysts, indicating the persistence of oxygen-containing functional groups associated with the lignocellulosic structure of the precursor material. The overall spectral features suggest that structural modifications during reuse are subtle and likely related to surface interactions^100^.

Based on these observations, the decline in catalytic activity is more reasonably attributed to the combined effects of (i) gradual leaching of acidic functional groups and (ii) adsorption or accumulation of reactants, intermediates, or products on the catalyst surface, which can limit access to active sites. Similar deactivation pathways have been reported for biomass-derived sulfonated catalysts used in esterification and transesterification processes^37,46^.

A more pronounced decrease in conversion was observed in later cycles, with values dropping to 37.3%, 14.4%, and 3.6% in the fourth, fifth, and sixth cycles, respectively. This behaviour highlights the limited reusability of the catalyst under the present conditions and indicates the need for improved stability. To enhance catalyst durability, regeneration strategies such as solvent or alkaline washing may help remove adsorbed species and partially restore catalytic performance^101,102^. In addition, future work will focus on catalyst regeneration and detailed characterization to better understand structural changes during reuse and to improve catalyst stability for practical applications.

### Mechanistic insight into one-pot hydrothermal in-situ sulfonation

In the one-pot hydrothermal carbonization and sulfonation route applied in this study, concentrated sulfuric acid acts simultaneously as the reaction medium and the sulfonating agent, and the synthesis mechanism is governed by the chemistry of hydrothermal conditions. Under subcritical temperatures in the range of 160–230 °C, biomass-derived precursors undergo dehydration and condensation reactions that generate reactive intermediates such as furfural and 5-hydroxymethylfurfural (5-HMF). These intermediates possess partially conjugated structures that polymerize and initiate carbon framework formation prior to complete aromatic condensation. In the presence of sulfonating species, electrophilic substitution reactions can occur concurrently with carbon network development, enabling in-situ incorporation of sulfonic acid (-SO₃H) groups into the forming carbon matrix. Similar mechanisms for simultaneous carbonization and sulfonation under hydrothermal conditions have been reported for biomass-derived solid acid catalysts^16,21,103,104^.

The formation of chemically incorporated sulfonic acid groups in the present catalyst is strongly supported by acid value determination. The total acidity increased from 0.66 mmol g⁻¹ for raw corncob to 1.793 mmol g⁻¹ after sulfonation, with -SO₃H groups contributing 1.103 mmol g⁻¹. This substantial increase in Brønsted acidity provides quantitative evidence of covalently bound sulfonic acid sites rather than physically adsorbed sulfur species, as weakly bound sulfate species do not contribute consistently to titratable acidity. Similar relationships between acid density determined by titration and catalytic activity have been reported for sulfonated biomass-derived catalysts used in biodiesel esterification and transesterification^46,47^.

FTIR analysis further confirms the chemical incorporation of sulfonic acid groups as shown in Fig. 3. The appearance of characteristic absorption bands in the 1200–1250 cm⁻¹ region, assigned to symmetric and asymmetric stretching vibrations of S = O bonds, is a clear signature of -SO₃H functionalities. The simultaneous decrease in the intensity of the broad -OH stretching band after sulfonation indicates the participation of surface hydroxyl groups in the sulfonation reaction, consistent with covalent grafting mechanisms. These FTIR features are widely accepted as evidence of chemically bonded sulfonic acid groups in sulfonated carbon catalysts rather than physiosorbed sulfur species^17,105^.

Additional confirmation is provided by SEM-EDX analysis as shown in Fig. 4. The sulfonated corncob exhibits a denser and smoother carbon morphology, which is characteristic of surface modification and partial pore filling by grafted functional groups. EDX analysis confirms the presence of sulfur (1.09 wt%), uniformly distributed within the catalyst matrix. This sulfur content falls within the typical range reported for effective sulfonated lignocellulosic carbons and is sufficient to generate strong Brønsted acidity for esterification and transesterification reactions. The combined presence of sulfur detected by EDX, sulfonic acid bands in FTIR spectra, and titrated acid density provides converging evidence for covalent incorporation of -SO₃H groups into the carbon framework^16,21,106^.

From a process design perspective, the one-pot synthesis strategy offers several important advantages. It simplifies the overall preparation route by combining carbonization and sulfonation into a single step, thereby reducing the number of processing stages and lowering overall energy demand compared with conventional two-step methods. Because the acid functions both as the reaction medium and the sulfonating agent, the amount of additional hazardous chemicals required is also reduced. Furthermore, carrying out the reaction under hydrothermal conditions is consistent with the principles of green and sustainable chemistry, as multiple transformations are integrated within a single reaction environment. These characteristics make the one-pot approach particularly well suited for early-stage life cycle assessment evaluations, allowing environmental benchmarking of solid acid catalysts at an early stage of development^16,104^.

Nevertheless, inherent limitations arise from the hydrothermal one-pot approach. The carbon framework formed under hydrothermal conditions is generally less condensed and less aromatic than materials produced via high-temperature carbonization, which can limit the density and stability of sulfonic acid groups. As a result, catalysts synthesized through this route often exhibit lower sulfur content and reduced stability of acidic sites upon reuse. The observed decline in catalytic activity after repeated cycles in this study is consistent with these mechanistic constraints and reflects an accepted trade-off between catalyst durability and reduced process severity^17,47,103,104^.

### LCA results and discussion

The discussion compares all experimental runs to identify the most environmentally sustainable conditions. Electricity and solvent use during catalyst synthesis, oil extraction, and esterification/transesterification were the main hotspots. Under optimized conditions, impacts on climate change, fossil resource depletion, and human toxicity were markedly reduced. Three-dimensional response surface plots (Figs. 14, 15 and 16) reveal how catalyst loading, reaction temperature, and reaction time interact to influence environmental performance.

#### Environmental impact implications of process parameters

The absolute environmental impacts per kilogram of biodiesel appear relatively high because the assessment is based on laboratory-scale data, where energy and material consumption are not yet optimized. For this reason, the interpretation focuses primarily on relative differences among the experimental runs rather than on the absolute magnitude of the impacts. In addition, the zero-burden assumption applied to corn cob residues and DS seeds does not influence the comparative results, since all scenarios were evaluated using identical system boundaries, functional units, and allocation rules.

**Fig. 13:**
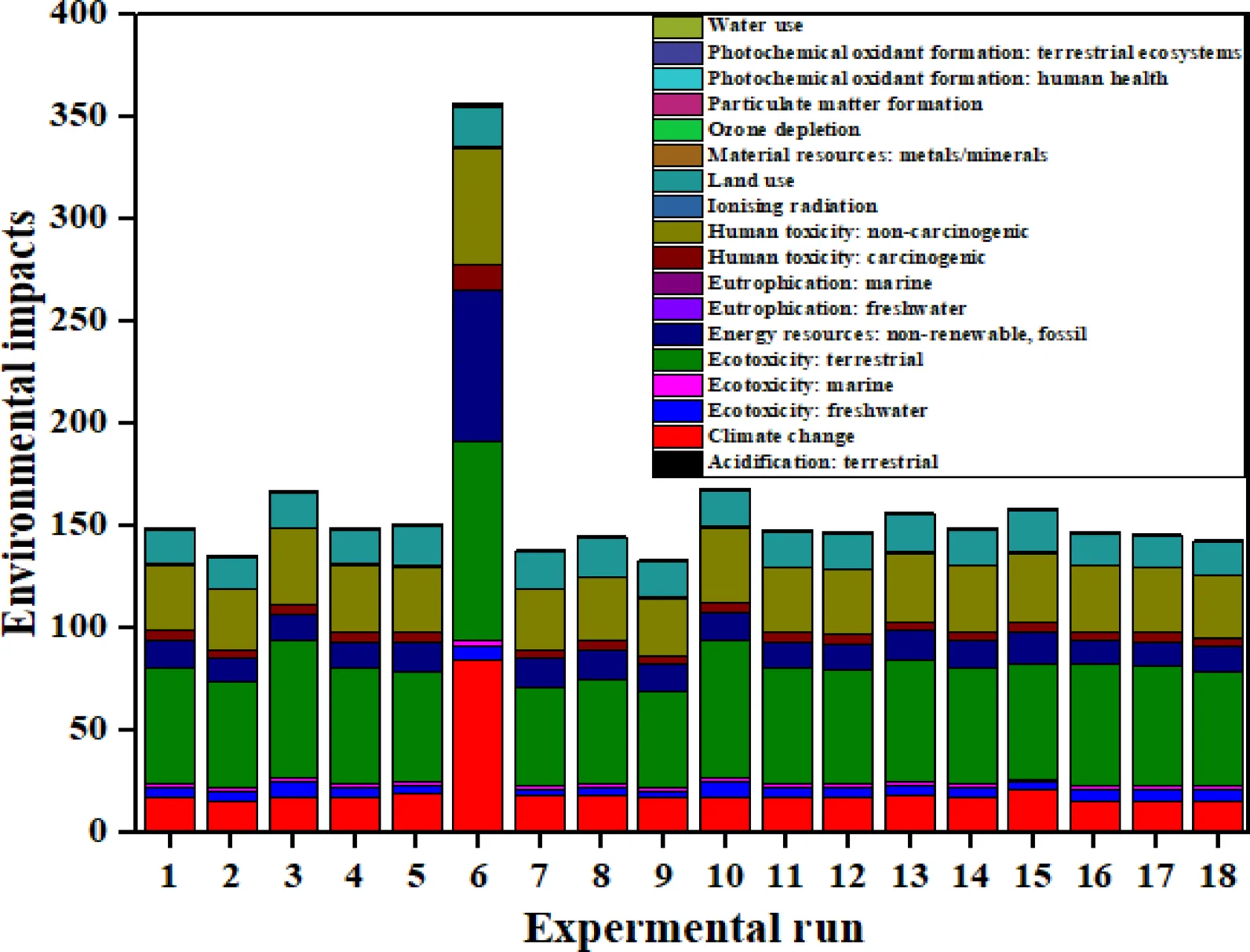
Environmental impact of each experimental run.

Figure 13 shows the effects of catalyst loading, reaction temperature, and reaction time on the environmental impacts of the 17 experimental runs and the optimal 18th run in one-pot, sulfonated corn cob solid acid-catalysed biodiesel production from DS seed oil. Biodiesel conversion varied from 64.0% (Run 15) to 89.7% (Run 17), with higher catalyst loadings (7–10 wt%) and moderate temperatures of 55 °C favouring better esterification/transesterification. On the contrary, incomplete conversion was observed to occur either at extreme temperatures, such as 65 °C (Run 6) or due to insufficient reaction times. Based on LCA, the leading contributors to environmental burdens were including the energy-intensive operation (electricity for heating), methanol use and evaporation in esterification, hexane consumption and loss during oil extraction, and the use of sulfuric acid for the catalyst synthesis. Climate change potential varied greatly in the range from 15.40 kg CO₂-eq for Run 17 up to 84.75 kg CO₂-eq for Run 6, whereas acidification varied from 0.126 to 0.239 kg SO₂-eq as shown in Supplementary Table 2(ST2). The wide global warming potential (GWP) ranges from approximately 15 to 84 kg CO₂-eq per kg biodiesel largely reflects the nature of laboratory-scale experimentation and the specific assumptions used in the life cycle assessment rather than the performance of an industrial biodiesel process. At small scale, low oil-to-biodiesel conversion and unconverted oil losses mean that a similar amount of electricity, heat, solvents, and chemicals is consumed but less product is obtained, so when these inputs are normalized to 1 kg of biodiesel the resulting emissions appear elevated; industrial systems benefit from higher conversions, co-product allocation and scale efficiencies, with recent biodiesel LCA studies reporting benchmark GWPs on the order of 2.63–2.88 t CO₂-eq per tonne biodiesel (equivalent to 2.6–2.9 kg CO₂-eq/kg) for large and small-scale rapeseed biodiesel when agriculture and processing are included, illustrating how scale and yield affect results^107^. In our laboratory context, electricity consumption for prolonged heating in Soxhlet hexane extraction, hydrothermal one-pot catalyst synthesis, and esterification/transesterification contributes disproportionately to GWP because laboratory equipment lacks heat integration and energy efficiency, a pattern also observed in small-scale extraction LCAs where heating demands drive environmental impacts^108^. The use of solvents such as hexane for oil extraction and sulfuric acid in catalyst synthesis further increases impacts, consistent with findings that solvent intensity is an important determinant of environmental performance in biodiesel pathways^109^. Methodological choices including mass-based allocation of catalyst synthesis impacts across multiple runs and cut-off rules that exclude upstream agricultural and transportation burdens for locally sourced biomass also affect the absolute magnitude of the GWP range and would differ in a fully expanded industrial boundary. Consequently, runs with inefficient conversion but similar energy and chemical consumption display higher GWP, whereas runs with relatively higher conversion under moderate energy and chemical use yield lower impacts. These results underscore the influence of laboratory-scale inefficiencies, energy-intensive solvent and electricity use, allocation methods, and boundary definitions on GWP outcomes. They also highlight that this LCA is intended for comparative evaluation of process conditions rather than to predict optimized industrial biodiesel production performance. Freshwater and marine ecotoxicity metrics ranged between 3.22 and 6.85 kg 1,4-DCB-eq and followed a similar trend, indicating that those metrics quantify the accumulation of unreacted triglycerides and solvent residues and wastes from runs characterized by low conversion. These results indicate that poor reaction conditions enhance environmental impacts and increase resource consumption. This confirms earlier and recent LCA findings for biodiesel systems, emphasizing the key role of process energy intensity and chemical consumption as the driving force for environmental burdens^110,111^.

When the climate change impacts obtained in the present study are compared with those reported in recent biodiesel life cycle assessment literature, clear insights emerge regarding the influence of catalyst performance, conversion efficiency, and laboratory-scale system boundaries. In this laboratory-scale LCA, catalyst reuse was not explicitly credited in the impact calculation, although the reusability of the synthesized sulfonated biomass-based catalyst was experimentally demonstrated for at least four consecutive cycles, as shown in Fig. 12. Despite this demonstrated reuse potential, the environmental burdens associated with catalyst synthesis were conservatively allocated to biodiesel production without accounting for multiple reuse cycles. As a result, the reported climate change impacts represent a worst-case scenario, in which the full carbon burden of catalyst preparation contributes to the calculated global warming potential. Under these assumptions, the climate change impacts across the evaluated experimental runs ranged from approximately 15.4 to 84.7 kg CO₂-eq per kg biodiesel, with the optimized condition achieving 15.7 kg CO₂-eq per kg biodiesel at 91.5% conversion as shown in Supplementary Table 2(ST2). Although these values are substantially higher than typical industrial biodiesel benchmarks, they are consistent with laboratory-scale assessments where low conversion efficiency, solvent-based oil extraction, and energy-intensive heating substantially inflate impacts when normalized to 1 kg biodiesel. Comparable trends are reported in the literature. For example, Ao et al. (2025) reported a climate change impact of 4103.49 kg CO₂-eq per 1000 kg biodiesel (approximately 4.1 kg CO₂-eq per kg biodiesel) using a superhydrophobic biochar catalyst derived from *Citrus sinensis*, clearly demonstrating the combined benefits of high conversion efficiency and catalyst reuse^112^. Similarly, Ao et al. (2024) reported a global warming potential of 3.17 kg CO₂-eq per kg biodiesel for an active-sites-engineered biomass-carbon catalyst achieving nearly 97% yield, highlighting how optimized catalyst design and effective utilization markedly reduce carbon footprint^113^. Beyond these catalyst-focused studies, broader biodiesel LCA research consistently identifies energy demand and process scale as dominant contributors to climate change impacts. In particular, laboratory-scale systems are highly sensitive to electricity consumption for heating and solvent-based oil extraction, where the absence of heat recovery and process integration leads to disproportionately high emissions per unit of biodiesel^114^. These findings align closely with the present results, where experimental runs with lower oil-to-biodiesel conversion exhibit markedly higher CO₂ emissions despite similar material and energy inputs. Conversely, higher conversion runs with moderate energy and chemical consumption show substantially lower climate change impacts. Importantly, if catalyst reuse were formally incorporated into the LCA, even conservatively assuming four reuse cycles as demonstrated experimentally in Fig. 12, the contribution of catalyst synthesis to total climate change impact would be significantly diluted. Therefore, while the absolute GWP values reported in this study are not representative of commercial biodiesel production, the observed trends are fully consistent with established LCA literature and confirm that conversion efficiency, energy intensity, and catalyst utilization strategy are the primary determinants of the carbon footprint in biodiesel production systems.

The three-dimensional (3D) response surfaces were developed to visualize the interaction effects among catalyst loading, temperature, and reaction time for the major environmental categories, including acidification, climate change, and freshwater ecotoxicity. Only the 3D surfaces for acidification, climate change, and freshwater ecotoxicity impact categories are presented here for clarity, while the complete environmental impact dataset is provided in the Supplementary Material Table 2(ST2).

**Fig. 14:**
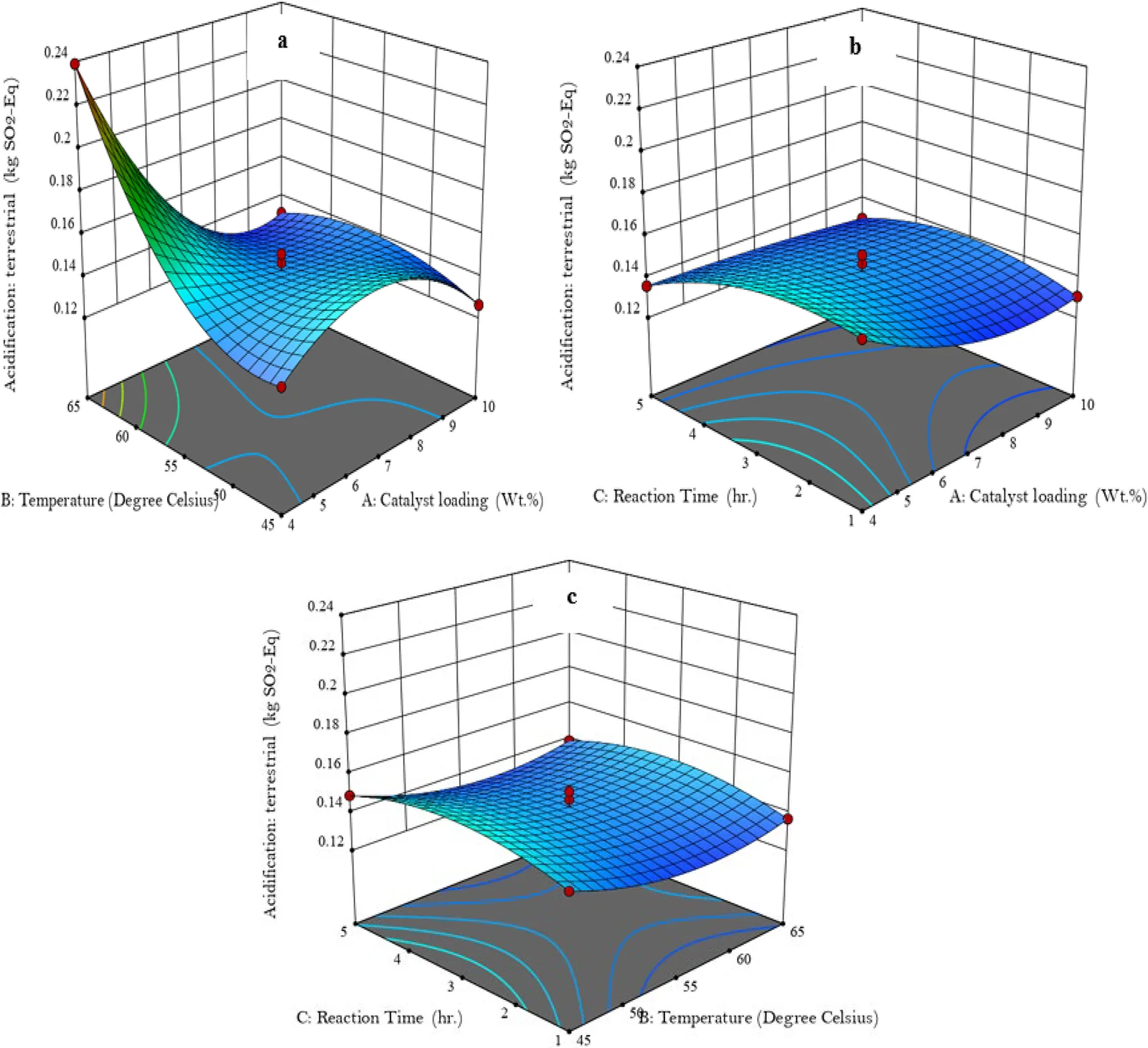
Three-dimensional response surface plots illustrating the influence of process parameters on terrestrial acidification impact: **a** temperature vs. catalyst loading, **b** reaction time vs. catalyst loading, and **c** reaction time vs. temperature.

Looking at terrestrial acidification in the biodiesel production system presented in Fig. 14, it can be observed that high temperature-low catalyst loading combinations resulted in the most significant acidification values (up to 0.239 kg SO₂-eq, run 6), while the highest catalyst loading at moderate temperatures reduced acidification to the minimum value recorded (Run 17: 0.129 kg SO₂-eq). The latter trend follows the impact of catalyst performance on completeness of reaction and associated electricity demand for heating and solvent recovery, in line with recent LCAs underlining the role of catalyst performance and thermal management as main drivers of acidification burdens^15,113^. When considering catalyst loading and reaction time at the centre-point temperature (55 °C), low catalyst loadings paired with short reaction times (Run 15: 0.157 kg SO₂-eq) increased acidification due to poor triglycerides conversion and increased auxiliary energy use, while catalyst loading increase was found to decrease acidification, even at short reaction times (Run 9: 0.130 kg SO₂-eq), thus confirming the paramount role played by catalyst mass in minimizing SO₂-equivalent emissions thanks to improved conversion kinetics and reduced needs for downstream processing^110^. The interaction between temperature and reaction time at a fixed catalyst loading (7 wt%) revealed that short reaction times at elevated temperatures (Runs 12 & 14: 0.136–0.138 kg SO₂-eq) led to moderate acidification, while prolonged times at moderate temperatures (Run 13: 0.1467 kg SO₂-eq) slightly increased acidification due to additional heating and mixing energy, indicating that excessively high temperatures or long reaction durations offer diminishing returns and may raise auxiliary energy consumption, in agreement with broader LCA studies emphasizing careful optimization of the temperature-time interplay to achieve minimal acidification while maintaining efficient conversion^111^.

**Fig. 15:**
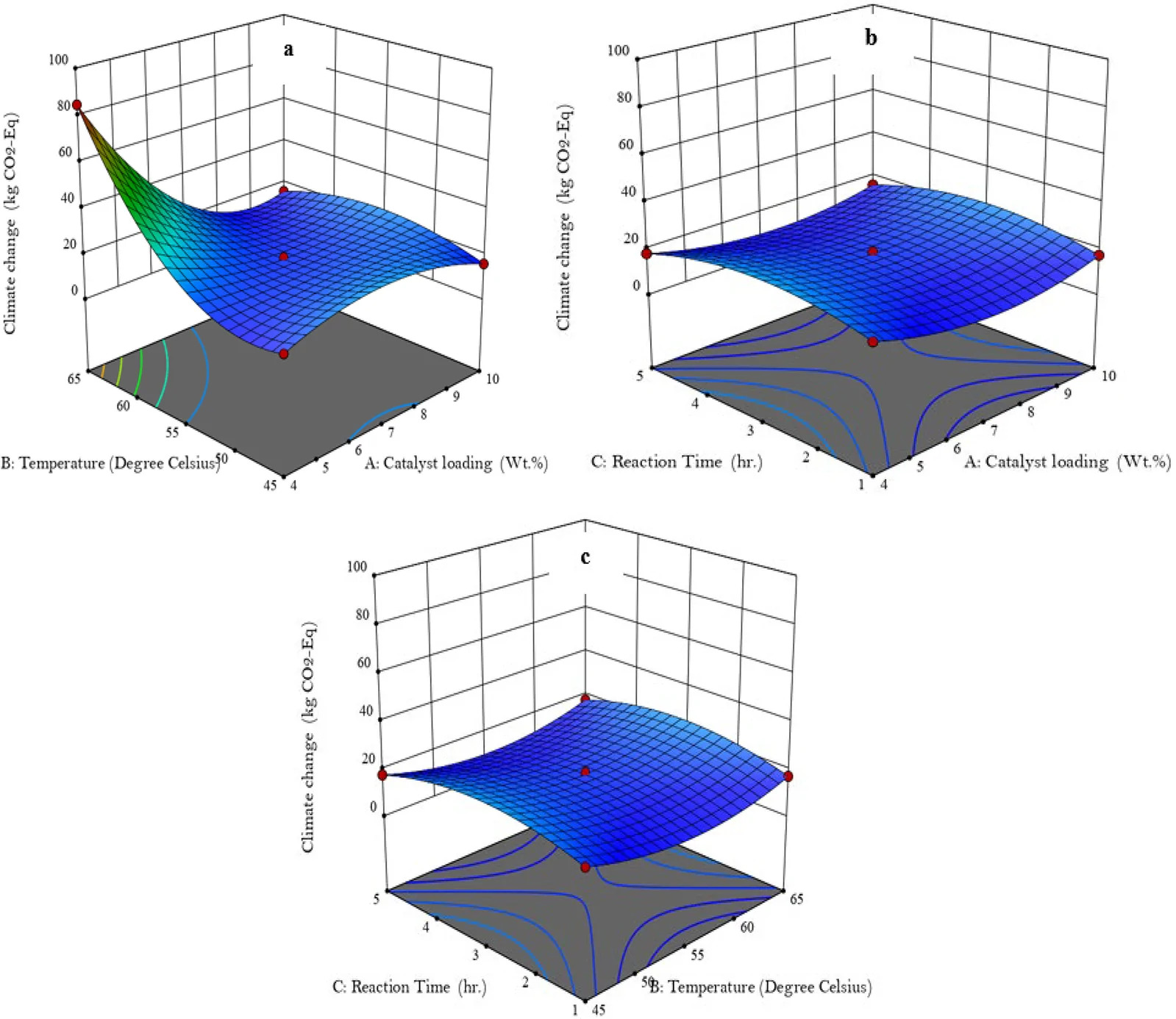
Three-dimensional response surface plots illustrating the effects of process parameters on climate change impact: **a** temperature vs. catalyst loading, **b** reaction time vs. catalyst loading, and **c** reaction time vs. temperature.

The climate warming potential (GWP) of biodiesel production had a pronounced sensitivity related to temperature and catalyst loading. CO₂-equivalent emissions rose sharply to 84.7 kg CO₂-eq due to the high temperature (65 °C) combined with low catalyst loadings because of low conversion, extended heating periods, and excessive energy input, while higher catalyst loadings (9–10 wt%) at moderate temperatures (55 °C) minimized the emissions to 15.4 kg CO₂-eq as shown in Fig. 15. This agrees with studies that show how optimized heterogeneous catalysis and energy-efficient process conditions contribute to substantial reduction in the global warming potential^115–117^. The interaction between catalyst loading and reaction time at the centre-point temperature of 55 °C revealed that low catalyst loadings (4–5 wt %) combined with extended reaction times of 1–5 h yielded only a modest improvement in the process efficiency while keeping GWP high at 20.66 kg CO₂-eq due to poor catalyst utilization and long heating. In contrast, the higher catalyst loading within 7–10 wt% accelerated the esterification/transesterification kinetics and dropped GWP to 15.4–17.8 kg CO₂-eq within reasonable reaction times, which reflects the role of adequate catalyst mass in reducing life-cycle emissions^110,111^. Similarly, the interaction between temperature and reaction time at a fixed catalyst loading of 7 wt% indicated that a shorter reaction at an elevated temperature (65 °C, 1 h) led to a moderate environmental performance of 17.07 kg CO₂-eq due to its high energy input. In turn, longer reaction times of 5 h at a moderate temperature of 55 °C reached near-optimal environmental outcomes of 15.4–17.8 kg CO₂-eq without excess emission. Thus, excessive temperature or prolonged heating increases the energetic penalty and GWP, and the reaction time must be balanced against temperature to achieve environmentally sustainable biodiesel production^111,118^.

**Fig. 16:**
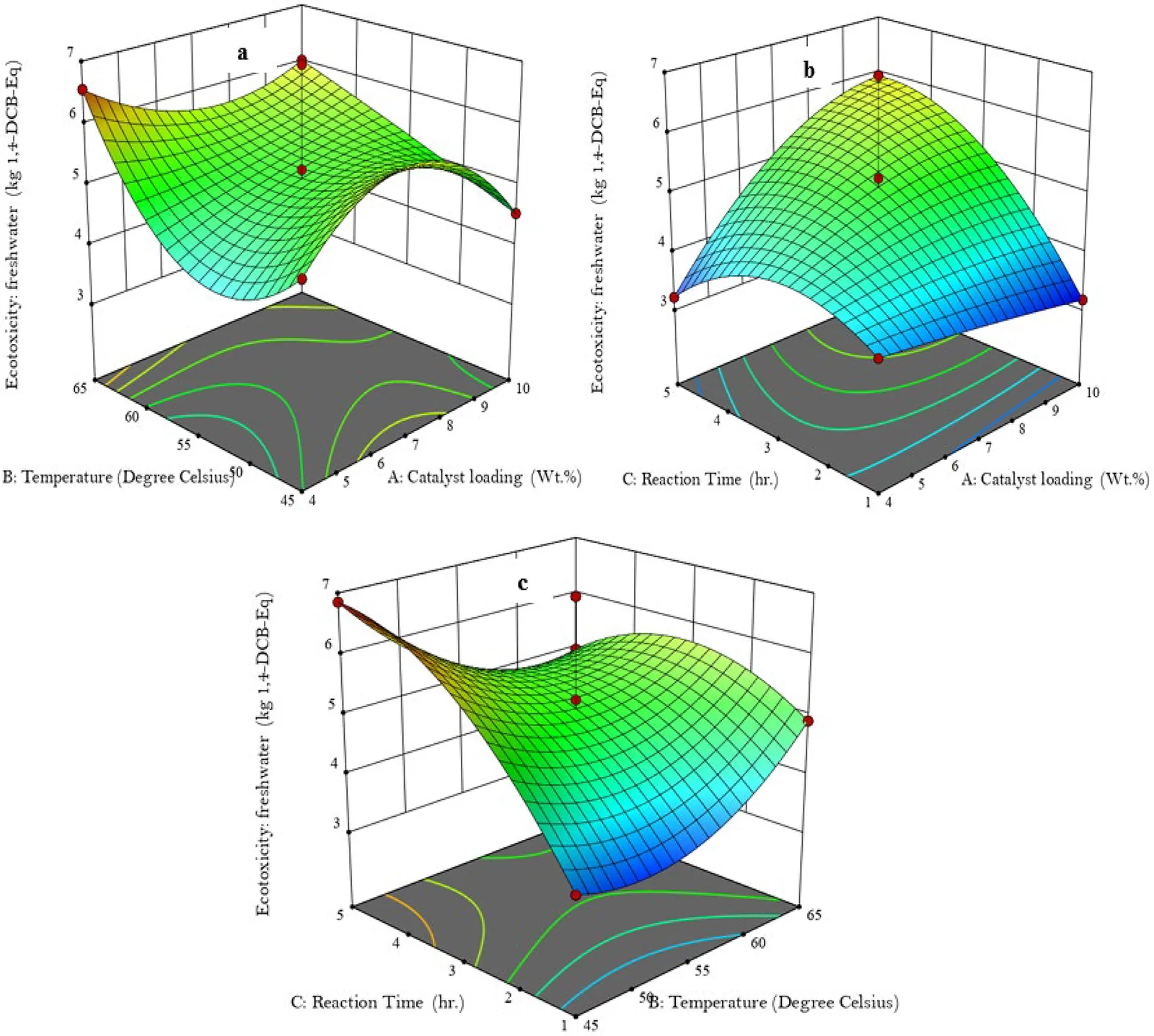
Three-dimensional response surface plots depicting the influence of process parameters on freshwater ecotoxicity impact: **a** temperature vs. catalyst loading, **b** reaction time vs. catalyst loading, and **c** reaction time vs. temperature.

The freshwater ecotoxicity results showed that impacts were maximized under adverse interactions between catalyst loading and temperature, increasing up to 6.55–6.85 kg 1,4-DCB-eq as shown in Fig. 16 at low levels of catalyst loading combined with high temperatures, conditions which impede catalytic performance and enhance methanol evaporation as well as overall solvent-related emissions during esterification/transesterification at constant time at the center point (3 h). In contrast, medium to high catalyst loadings of 7–10 wt% combined with more balanced thermal conditions drastically decreased the ecotoxicity to 3.81–3.95 kg 1,4-DCB-eq, indicative of more favorable methanol use and reduced losses with lower solvent intensities. This observation is consistent with recent LCA studies indicating that optimized catalyst performances coupled with controlled reaction temperatures significantly reduce aquatic toxicity in biodiesel systems^31,119,120^. At the centre-point temperature (55 °C), the interaction between catalyst loading and reaction time further showed that low catalyst input (4 wt%) produced impacts within the range of 3.82–4.93 kg 1,4-DCB-eq, while raising the catalyst to 10 wt% caused a reduction in ecotoxicity down to 3.18 kg 1,4-DCB-eq even at the shortest reaction times, once again confirming that catalyst mass is more effective in suppressing freshwater toxicity than prolonged residence times due to the reduced residual methanol and solvent release^111^. A complementary trend in the interaction between temperature-time showed that high temperatures of 65 °C with short reaction times of 1 h produced high ecotoxicity at 6.55 kg 1,4-DCB-eq, whereas moderate temperatures from 55 to 63 °C, at longer reaction times of 4–5 h reduced the impacts to 3.81 kg 1,4-DCB-eq, emphasizing that controlled heating combined with sufficient reaction duration promotes complete conversion and limits the release of solvents. This finding is further supported by evidence that balanced thermal profiles and adequate residence times decrease the toxic outputs in aqueous environments for biodiesel pathways^31,121^.

Overall, the multi-parameter interactions depicted by the 3D response surfaces showed that the catalyst loading and reaction temperature constituted the fundamental driving forces not only for technical performance but also for environmental outcomes, while reaction time was the secondary amplifier when excessive energy input was applied over kinetic requirements. The one-pot sulfonated corn-cob catalyst demonstrated superior environmental performance under through decreased consumption of concentrated sulfuric acid, minimizing wastewater generation, and energy-related emissions when compared with multi-step or homogeneous alternatives^19,122^. These findings reaffirm that integrating process optimization and catalyst circularity enhances the sustainability of biodiesel production by coupling high conversion with minimal environmental impact^61,113,116^.

#### Comparative environmental impact evaluation of the three critical run

**Fig. 17:**
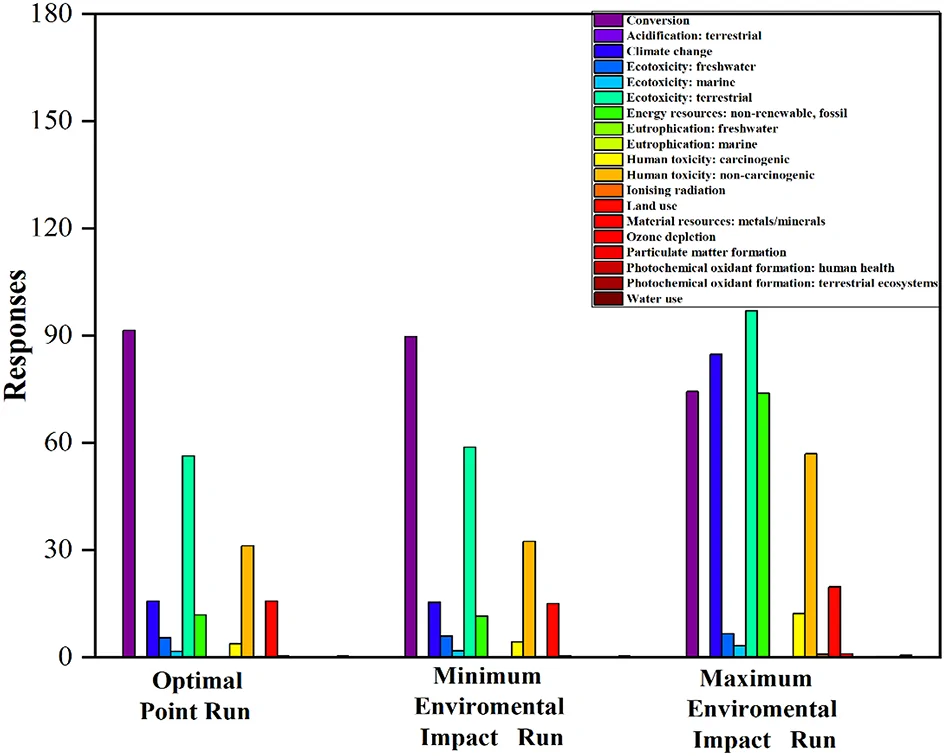
Comparative environmental impact evaluations of three critical runs.

Figure 17 presents a comparative assessment of the optimal, minimal, and maximal environmental impact runs, offering useful insight into the trade-offs between process efficiency and sustainability in biodiesel production from DS seed oil using a sulfonated corncob-based solid acid catalyst. The optimal run, which achieves the highest biodiesel conversion (91.35%), demonstrates strong catalytic performance; however, it is associated with slightly higher environmental burdens, including terrestrial acidification (0.1306 kg SO₂-eq), climate change potential (15.70 kg CO₂-eq), and fossil resource use (11.84 kg oil-eq), compared with the minimum-impact run. In contrast, the minimum-impact run, with a slightly lower conversion efficiency (89.7%), consistently shows reduced emissions across most impact categories, indicating a better balance between process performance and environmental sustainability. Conversely, the maximum-impact run (74.3% conversion) shows substantially elevated impacts, including climate change (84.75 kg CO₂-eq), fossil depletion (73.83 kg oil-eq), and terrestrial ecotoxicity (96.93 kg 1,4-DCB-eq), indicating inefficient reaction performance and higher energy and material consumption. These trends are consistent with previous LCA-based biodiesel studies, where higher conversion does not necessarily correspond to lower environmental burdens due to the influence of catalyst preparation, solvent usage, and energy demand^9,31,110^. Similar observations have been reported for biomass-derived catalysts, where process optimization plays a critical role in minimizing emissions while maintaining acceptable yields^39,123,124^.

When compared with recent literature, Burmana et al. (2025) reported a substantially lower global warming potential of about 1.04 kg CO₂-eq per kg biodiesel, mainly due to narrower system boundaries and a more optimized process configuration, including the reuse of by-products via acidification and extended catalyst reusability, which collectively reduced both material demand and energy consumption^125^. In contrast, the present study adopts a more comprehensive life cycle perspective that integrates catalyst synthesis, oil extraction, and fuel production under a conservative worst-case assumption of single-use catalyst operation, which inevitably increases cumulative environmental burdens. This broader system framing, together with limited heat integration and the inclusion of upstream and catalyst preparation stages, explains the relatively higher impacts observed here and highlights how methodological choices in boundary definition and process assumptions can strongly influence reported environmental performance.

Furthermore, Conventional fossil diesel exhibits life-cycle greenhouse gas emissions of approximately 90 g CO₂-eq/MJ (90 kg CO₂-eq/GJ), as reported in a peer-reviewed life cycle assessment study of transportation fuels^126^, indicating that biodiesel pathways still offer environmental advantages despite process inefficiencies. However, it is important to note that scaling the inventory from 0.0085 kg to a functional unit of 1 kg introduces uncertainty, as energy consumption and material flows do not scale linearly. Similar limitations have been acknowledged in recent LCA studies, where laboratory-scale data may not fully capture the efficiencies of industrial systems^127,128^. Therefore, while the present results provide meaningful comparative insights, they should be interpreted with consideration of these scaling limitations. Overall, the findings confirm that optimizing reaction conditions can reduce environmental burdens without substantial loss in conversion, supporting the development of more sustainable biodiesel production pathways^115,129^.

## Conclusion

This study demonstrated the successful synthesis of a one-pot hydrothermally sulfonated corncob catalyst and its effective application in the esterification/transesterification of high-FFA DS seed oil, attaining a validated biodiesel conversion of 91.35% under optimized conditions (8.75 wt% catalyst loading, 63.2 °C, and 4.8 h). Structural and thermal characterization confirmed the successful incorporation of -SO₃H groups and demonstrated that the catalyst can be reused for at least five cycles. Integration of LCA with experimental results indicated large variability in the environmental performance for the 17 runs: the poorly performing conditions resulted in a high impact because of the inefficient conversion, besides additional use of energy and solvent, whereas optimal operation minimized those energy and solvent losses, thus providing a favorable balance between biodiesel yield and sustainability. Accordingly, the optimum run provided 15.70 kg CO_2_-eq for climate change, 0.131 kg SO_2_-eq for terrestrial acidification, and 5.47 kg 1,4-DCB-eq for freshwater ecotoxicity, proving that appropriate combinations of catalyst loadings, temperature, and reaction time can enhance conversion while reducing environmental burdens simultaneously. While the use of agricultural residues for the catalyst synthesis and non-edible DS seed oil support circular bioeconomy principle, hotspots remain about methanol, hexane, and sulfuric acid production and heating energy. Further environmental performance improvements can be achieved through catalyst regeneration and reuse, replacing solvent extraction with mechanical pressing, and valorisation of seed meal and crude glycerol. Future LCA studies should incorporate catalyst regeneration and reuse and co-product substitution credits for a more comprehensive sustainability assessment.

## Supplementary Information

Below is the link to the electronic supplementary material.

Supplementary Material 1.

Supplementary Material 2.

## Data Availability

All data are included in this manuscript.
